# "The Hospital" Nursing Mirror

**Published:** 1900-10-06

**Authors:** 


### "The Hospital" Nursing Mirror.

Being the Nursing Section of "The Hospital."

[Contributions for this Section of "The Hospital" should be addressed to the Editor, "The Hospital" Nursing Mirror, 28 & 29 Southampton Street, Strand, London, W.C.]

### Notes on News from the Nursing World.

### AN INCENTIVE TO ASSIST IN OUR CHRISTMAS DISTRIBUTION.

There is every indication that this year the need for the distribution of warm clothing which we are able, through the thoughtfulness and generosity of our readers, to make at Christmas to the hospitals for the benefit of patients will be specially felt. We have already had applications from deserving institutions which have not so far participated in the gifts, and we hope to be able to respond to their pathetic appeals. One secretary writes: "In this poor neighbourhood our needs are never ending, and a little help towards brightening the Christmas of at least those patients then on the books would be most gratefully received." Each parcel, we remind the kind friends who intend to join in the good work, should have the name and address of the sender inside, so that we can acknowledge the contribution in our columns. The parcels should be addressed to "The Editor, *The Hospital*, 28 and 29 Southampton Street, Strand, London, W.C.," and should be inscribed "Clothing Distribution." They should reach us not later than Monday, December 17th.

### THE NEW COMMANDER-IN-CHIEF AND THE NURSES.

Among the nurses of the Imperial Yeomanry Branch Hospital at Eastwood, Pretoria, are those on the private nursing staff of the Royal Berkshire Hospital. One of these sends a letter to the Matron at Reading, giving an account of the visit of Lord and Lady Roberts to the hospital:—

It is with great pride I write the above address—our own hospital, where we are really settled at last. You will have seen in the papers that Lady Roberts opened it formally, and pronounced everything perfect. I think we were all very honoured to have both Lord and Lady Roberts come with their daughter, also General French, besides lots of others. We all had a very hard week getting ready; but when we think of what was accomplished, no wonder, for except blankets on some of the beds we practically prepared for 300 beds; 60 up at the house, where they are really beautiful little wards, different sizes of course; the largest downstairs, originally the dining and drawing-room, with folding doors. There are 18 beds, and the Sister who has that ward, a very pretty one, has gone on duty to-day. At present there are three rows of double tents, marquees, which hold 12 in each, and four rows of single marquees of six in each. Every two rows has a little bell tent for the two Sisters. We shall have 24 patients each in single tents. The first thing Lady Roberts did after her arrival was to present each one of us as we were introduced by Major Kilkelly, our commandant, with the I.Y.H. medal, which has the Prince of Wales's feather and scroll, on a rosette. After that they went round and inspected the wards; had tea in the conservatory, which I helped to get ready, do the flowers, &c. When they departed Lord Roberts rode behind the carriage between two Bengal Lancers with his bodyguard behind.

### NURSING THE BOERS.

A Scotch Sister at the Red Cross Hospital, Kroonstadt, writes us:—"I have nursed the Boers and am pleased to be able to say that I have found them courteous, obliging, and always grateful for everything that is done for them. Even when very ill and suffering great pain, I have never heard them grumble. If convalescent, they are willing to help those who are not so fortunate as themselves, and, at all times, make themselves agreeable to those in the hospital whom they come in contact with. Our soldiers and the Boers converse amiably with each other, and they seem quite happy in each other's society."

### THE NURSING ARRANGEMENTS IN SOUTH AFRICA.

A correspondent whose nursing experience is unquestionable sends us a letter on the nursing arrangements in South Africa, which points to the conclusion that there is great room for reform in the Army service. Herself attached when she is at home to the staff of a leading English hospital, she freely admits that many of the sisters sent out are most excellent private nurses, or most useful in a place where they have someone over them, but she says that some of them "are no more capable of being placed in charge of a large number of patients than a junior teacher in a high school could be if she were suddenly promoted to be head mistress." It will be observed that she does not raise the question of training. Her contention is that the three years' certificate is not the one essential qualification, but that "capable, steady women with experience of ward management or superintendence in a London or great provincial hospital, should be chosen and placed in charge of a division, or so many wards, and that they should be responsible that things are properly done, their standing to be somewhat the same as that of a night sister at a large hospital." This she contends is necessary, because it is impossible that the Army Superintending Sister, on whose shoulders falls the housekeeping, can find time to go in each ward every day even for a hurried visit. Certainly the picture of a girl who has only just finished her training managing fifty patients, "to say nothing of orderlies," is not one that will commend itself to those who desire that the nursing arrangements under the direction of the War Office should be the very best that can be devised.

### FRICTION BETWEEN WORKHOUSE OFFICIALS.

The Matron of St. Pancras Workhouse, Miss Reckless, says she is quite sure that much of the friction between masters or matrons of workhouses and infirmary nurses might easily be prevented by the exercise of a little tact and common-sense. Perhaps—as sometimes happens, particularly in small workhouses—the matron may have risen from the rank of porteress, and the nurse may be a lady by birth; it is here that the shoe pinches. "But," said Miss Reckless, "if the workhouse matron is broad-minded enough to acknowledge that nurses and nursing and all details of ward management are inseparable, and the superintendent nurse, in addition to her nursing certificate, has a fund of common-sense and tact, the position need never be an irksome one. The master and matron are really responsible under the 'Poor Law Orders' for the administrative department; but, given a capable managing superintendent nurse, the sensible matron will let her authority be merely nominal, and the sensible nurse will appreciate and not abuse her privileges." At any rate, Miss Reckless is quite determined that there shall be nothing unpleasant in the relations between workhouse matron and infirmary matron at St. Pancras, and she feels very indignant when she reads of workhouse scandals. Miss Reckless, though not a certificated nurse, fully recognises the value of the thorough training so often insisted upon in the NURSING MIRROR, and had some training at the London Hospital herself before entering on her important duties at St. Pancras.

### THE NEW INFIRMARY WARDS AT ST. PANCRAS.

THE guardians of St. Pancras, having found that the accommodation for the sick of the parish was insufficient, have converted the "infirmary wards" at Cook's Terrace, Pancras Road, into hospital wards. The infirmary proper at Highgate, which has 500 beds, could not be added to on account of space, and the newly-converted wards are those which have been in use for the aged and infirm inmates of the workhouse. Whether the arrangement will cramp the workhouse accommodation remains to be seen; at any rate, more space had to be provided for the sick of this enormous parish, by direction of the Local Government Board, and the advantage of having part of the hospital on the spot is evident, as patients frequently come in in too weak a state to be conveyed the two or three miles to Highgate. Miss Blake, the newly-appointed infirmary matron at Cook's Terrace, has just entered upon her duties; she has at present four sisters, twelve charge nurses, and fourteen assistant nurses, and more are to be appointed at once. There are eight large wards, two on each floor, and each floor is in charge of a sister. Every effort is being made to have all the arrangements of the most modern description. St. Pancras is the largest workhouse in London, and has 2,200 inmates.

### BARBADOES GENERAL HOSPITAL.

A COPY of the Barbadoes *Agricultural Reporter* of September 6th has been sent to us. It contains a long article in defence of the management of the Barbadoes General Hospital, with an appeal to us to note that Miss Cargill, the last head nurse, resigned for other reasons than because she found it impossible to work with the managing committee. The secretary and manager had already written us to this effect, but Dr. Law Gaskin, one of the visiting surgeons to the hospital, states definitely in the *Agricultural Reporter* that her resignation was due "(1) to the fact that she found the sole charge of the nursing staff too much for her strength, and (2) that for personal reasons quite disconnected with the hospital she desires to return to England." On this point it may be remarked that the publication of Miss Cargill's letter of resignation, which has not apparently been sent to the Barbadoes papers, might have prevented a good deal of misconception. Dr. Law Gaskin also says that "so harmonious have been the relations between Miss Cargill and the committees that she will not be required to refund the money from her passage from England to Barbadoes which was forfeited by her resigning before the expiration of the period for which she was engaged." This is satisfactory, so far as Miss Cargill is concerned. But another nurse who undertakes the duties Miss Cargill could not continue to discharge may likewise find them unduly onerous, especially if, as we are informed, they include a quantity of writing and book-work which usually devolves in hospitals upon another official. In the published minutes of the directors of the Barbadoes Hospital under date July 16th, 1899, there is the following passage:—"Attention was called to that part of the head nurse's (Miss Walker) letter of resignation, in which she states that she should regret to spoil her record by her position here, which, she states, must occur so long as the entire control of the nursing department is not vested in the head nurse." It is, at any rate, desirable that intending candidates, now or subsequently, for the post which Miss Walker and Miss Cargill found too much for them, should fully understand the nature of the obligations the head nurse at the Barbadoes General Hospital is expected to assume.

### KING'S COLLEGE HOSPITAL.

THE introduction of the electric light at King's College Hospital is much appreciated. The lamps fixed over the head of each bed, and the hand-lamp, which can be fixed at will, are found to be exceedingly useful. They can be carried at night without fear of a sudden glare being thrown into the eyes of the patient. The great want at King's now is a new and enlarged out-patients' department; and this Miss Monk hopes to see in the course of time. Discussing the question of four years' training, Miss Monk confesses that she feels strongly that an additional year in a nurse's training would be of immense advantage to her. It would allow, she says, more time for the development of the ethical side of her profession—and Miss Monk uses the word with caution, for she looks upon a nurse's work as not merely a profession, but as distinctly a woman's work. She would like a two years' probationership and a two years' staff appointment; and does not approve of the plan, in use in one or two hospitals, of allowing the fourth year to be spent in private nursing. The difficulty, in her opinion, is one of age. A woman, she points out, cannot enter upon her training until she is twenty-three, and seldom does so before twenty-six; the additional year would therefore mean that she would not be qualified to earn her living until the age of thirty. But Miss Monk holds that she would make a better nurse.

### NURSING IN PRISON.

THE account in another column of a night spent by a nurse in one of her Majesty's prisons claims the attention of the authorities, and we hope that the precedent set at Aylesbury Prison Infirmary by the appointment of Miss Wilson will be generally followed. It is very strange that it should be necessary in a public institution possessing an infirmary to send a warder out late at night to hunt up a nurse when one of the inmates is seized with sudden illness. Again, it may be essential that a nurse should be locked in a room with a prisoner all night, but it cannot be so that she herself should in other respects be treated as a prisoner. Our correspondent, who of course went without notice, at the call of duty, to stay the whole of Saturday night with a woman who needed her attention, was only provided with a hard wooden chair to sit upon, and was not offered any kind of food the whole of the night. It is not surprising that when just before seven o'clock on Sunday morning the wardress placed one eye at a tiny window in the door, and asked from outside if everything were "all right," the nurse felt "a distinct longing to throw a piece of coal at the eye." A less patient nurse would have yielded to the longing.

### "OPEN-AIR" BALCONIES.

The "open-air" balconies at the North London Consumption Hospital are well worth a visit. From the winter garden, looking bright with shrubs and scarlet geraniums, and furnished with comfortable substantial-looking armchairs, meant as a promenade and resting-place for the patients on wet days, one passes into the lower balcony, a long corridor, with open front, facing south-west. It has blinds of thick rain-proof material arranged to draw down should the rain beat in that direction; otherwise it is quite exposed to the weather. The floor is covered with thick linoleum, and there are strong military-looking couches covered with American cloth provided for patients who are considered "bed cases" and are not allowed to move about until their temperature becomes normal and their general condition improves. The balcony over this and leading from the men's corridor looks a very pleasant place with its row of twelve beds covered with scarlet counterpanes, and armchairs placed here and there with pots of flowers as decoration. Above is the women's balcony, holding twelve beds like the one below; it is covered with pretty pink cretonne counterpanes and tastefully decorated with plants, the patients having a magnificent view over the tree-tops towards the Surrey hills. Nursing in so much open air is found to be very beneficial, many nurses who look pale and colourless on arrival, improve in appearance and weight, and are apt to become somewhat of a trial to their friends by their insistence on open windows and their complaints of "stuffiness." The success of the balconies in the winter is yet to be told, but judging from former results on two small balconies there is not much to fear regarding the patients. The greatest trial of the nurses seems to be from chilblains, which may be due to the fact that they do not dry their hands sufficiently, and to their working in the keen, frosty air, or carrying hot bottles so constantly and thus getting a quick change of temperature. This condition, it is hoped, will be altered by giving the nurses warm woollen gloves and insisting on them being worn at all times excepting when their work renders this impossible.

### NURSE-DISPENSERS.

The directors of Arbroath Infirmary have decided "to lessen the expenditure of the institution" by appointing a nurse who is qualified to dispense, the period of dispensing being not more than two hours a day. It would be interesting to know the amount of the salary the directors intend to offer. Clearly, a nurse who, in addition to her ordinary training, has been at the expense of learning how to dispense, is entitled to demand a higher rate of payment. On the other hand, public bodies who are substituting female for male dispensers will have to be extremely careful not to entrust the work to any but fully qualified women. A fatal mistake by an unqualified female dispenser would have a disastrous effect upon the extension of a departure which may, in time, become a common practice.

### A FRIEND OF NURSES.

Five-and-twenty years ago, when genial "Harry Jones" was rector of St. George's in the East, he expressed himself satisfied that the trained nurse provided by the London Nursing Society was one of the most useful personages in his crowded parish; and though he did little begging, he often used to say that those who wanted to take part in "a distinctly good work could not do better than send a donation to the London Nursing Society." He was also one of the organisers of the East London Nursing Association. This excellent clergyman, we regret to learn, died on Sunday, and both the Church and the world are the poorer for his removal.

### A SCHEME FOR ABOLISHING THE PRIVATE NURSE.

It has been pointed out to us that if the provisions of the Workmen's Compensation Act were applied to nurses, the different institutions would guarantee persons employing their nurses immunity from litigation. No doubt they might. But what about the private nurse? The truth is that the extension of the Act to nurses would compel all the members of the profession to attach themselves to institutions. Sickness and the payment of a nurse are a sufficient expense in any case, and the average householder would certainly refuse to be made responsible for illness contracted by the nurse when she was under his roof.

### "A POOR-HOUSE NURSE ON HER DIGNITY."

Under this heading the *Leicester Post* of last week reports the following incident:—

"At a meeting of West Bromwich Board of Guardians, Mr. Blades in the chair, Miss Jacques, of Leicester, and Miss Skidmore, of London, were appointed night nurses at the infirmary. A deadlock, however, ensued, Miss Skidmore, owing to some insults that she alleged had been offered to her whilst in the board-room, declining to take the position. Efforts to prevail upon her to reconsider her decision were futile, and the board, having no alternative, gave the appointment to another candidate, Miss Hughes, of Worcester."

In the absence of particulars we do not, of course, know what transpired in the board-room at West Bromwich, but the allegations of Miss Skidmore are too serious to be passed over with a sneer. A "poor-house nurse" is as much entitled as any one else to have her feelings considered; and though the West Bromwich guardians may be incapable of offering an affront to a woman, the questions asked by guardians of some other boards are frequently objectionable.

### "AN UNFORTUNATE MISTAKE."

A demonstration was being held at one of the large London hospitals. The object of the lecture was a man of the nervous type—the subject his eyes. "You see, gentlemen," said the demonstrator, "that one pupil is dilated and the other contracted—an important thing to notice in determining which part of the brain is affected." The patient suddenly became aware that the discussion was upon his eyes. "Beggin' your pardon, sir," he blurted out, "but if it's my eyes you mean, one of 'em's a glaws 'un."

### SHORT ITEMS.

At the eighth annual meeting of the North Argyll Nursing Association it was reported that the staff consists of six nurses, who last year attended 472 patients. Unfortunately, the Association has a deficit of £29.—Three of the nurses trained at St. John's Hospital, Limerick, under the management of the Sisters of the Little Company of Mary, have passed their preliminary and final examinations in Anatomy and Physiology, Medical, Surgical, Fever, and Gynæcological Nursing. Two of the nurses passed with First Class Honours and one with Honours.—The Free Home for the Dying has been removed from 82 The Chase, Clapham, to the new and permanent Home at 29 North Side, Clapham Common, S.W.—The farewell meeting in connection with the departure of lady missionaries to India, under the auspices of the Zenana Bible and Medical Mission, will be held at Exeter Lower Hall on Tuesday afternoon at 3, Sir Charles Elliott, K.C.S.I., in the chair.

### Lectures on Medicine to Nurses.

By H. A. LATIMER, M.D. (Dunelm), M.R.C.S. Eng., L.S.A.Lond.; Consulting Surgeon, Swansea Hospital; Past President of the South Wales and Monmouthshire Branch of Brit. Med. Assoc.; Past President of the Swansea Medical Society; Hon. Life Member of the St. John Ambulance Association, &c.

### THE THERMOMETER AS A GUIDE TO DISEASE; VARIETIES OF FEVERS; THE PULSE.

A FEW words more on the subject of temperatures and I shall pass on from the consideration of general questions to that of particular diseases. I have warned you to be particular in setting the index of the thermometer to a point well below the normal one, for not only do low temperatures indicate depressed vital energy, but sudden falls in body-heat give warnings of complications which are about to ensue during the progress of illnesses. Thus, during an attack of enteric fever, if the temperature suddenly falls, the physician is on the look-out for an internal hæmorrhage, to which it is frequently the prelude; and a sub-normal temperature is often the precursor of death, at a time, too, when an unobservant attendant may think from an invalid's expressions that the latter is recovering from his illness. It is true that an opinion on an illness should not be based on one point alone, such as this of observing body-heat, and that to anyone who aims at being thorough in his work it is not so: but as we are now discussing the value of the use of the thermometer in disease I may speak of some of the indications it affords us by its use. It will suffice to illustrate what I have been saying by taking a fatal attack of peritonitis as an example. Here, if the illness is conducting to a disastrous ending, the fever and great pain which have been its prominent features are apt to be exchanged for a fallacious calm; the patient will tell you that he is quite comfortable, and is out of pain; his mind will be clear, and a superficial observer may think that he is on the high road to recovery; but it will be noticed that the pulse has become small and thready, and that the thermometer, placed in the rectum in such cases, marks a great fall of temperature. In such a case we are dealing with the condition called collapse, and we may be pretty sure death will shortly ensue. The moral of all I have been saying, then, is this: set the index of a thermometer below the normal point whenever you use it, so that it may register sub-normal temperatures as well as normal and febrile ones.

Now let me tell you what names are commonly applied to the various grades of fever-heat. A temperature of 101°·3 Fahr. is termed sub-febrile; one of 103° Fahr., moderate fever; one of 105° Fahr., severe fever; and when the register is 106° Fahr, and over, it is called hyperpyrexia, or excessive fever. You will note that the thermometers in this country are graduated according to the scale of Fahrenheit. On the Continent they are mostly according to the scale of Centigrade, in which the normal point of body-heat is about 37°. In some special cases you will be instructed to take four-hourly observations and to chart them. The only objections which can be urged to the use of the thermometer is that which applies in the cases of very nervous people, who are sometimes frightened into feverishness by it. You would hardly imagine that anxiety and terror could cause a rise of body heat, but, nevertheless, such may be the case. Every doctor has met with people who have suffered from excess or depression from such a cause.

The varieties of fevers are classified, according to their behaviour, largely on account of the heat which prevails during the attack, as well as on account of other symptoms. Thus we speak of a continued fever when the temperature maintains a more or less steady course; of an intermittent or remittent fever when the disease apparently subsides or slackens at certain intervals, to again appear; and of a relapsing fever when, after an apparent interval of recovery, all the former symptoms of illness are resumed. All these febrile affections have their origin in definite disturbances of the blood, induced for the most part by its infection with poison germs of one sort or another. We therefore term them specific fevers. Inflammations of organs are often accompanied by fever, but in such cases we call the resulting illness after the primary cause of it, and say that the person who is ill is suffering from inflammation of the brain, of the lung, or of whatever part may have originated the disturbance. The distinctions are artificial ones, made for the purpose of convenience; for in all cases fever is the result of blood infection, whether from poisons of the acute zymotic type, the malarial parasites which have been introduced into it from without, or from poisonous substances generated within the body by faulty actions of diseased organs there; indeed, as knowledge increases, we learn that even some of these fevers resulting from inflammation of organs within us are due to specific infection from without. There is little doubt, for instance, that pneumonia is often caused by an infective micro-organism. The lightest form of continued fever we meet with is simple continued fever—febricula as it is termed. As I shall begin my description of the fevers with this complaint I will not dwell upon it now. Up to the present I have said nothing to you about the pulse. This, in the old pre-thermometer days, was considered of paramount importance as a guide to the physician, and if he did not feel this carefully, with watch in hand, and if he omitted to look well at the tongue, his patient would hardly think that he had fairly earned his fee. The pulse is still a most valuable indication of health or disease in a person, but the mere routine counting of the number of its beats in a given space of time, as is often done, is of but very little use. You know what it is. The heart, in its contraction of the left ventricle, has sent a wave of blood into the arteries, and the impulse which you feel under your finger placed over an artery is this wave as it reaches the spot you are examining. Because the radial artery at the wrist is at a convenient situation for examination, this is the part usually selected for ascertaining the pulse-wave, and I need hardly warn you against thinking that a pulse only exists at this spot. As a matter of fact the examination can be made anywhere where an artery is superficially situated, so as to be readily accessible to the touch; and you will often see the physician place his finger, for this purpose, upon one of the temporal arteries which conduct blood to the forehead, across the temple, on each side of the head. It is necessary to feel some vessel which lies well exposed when one is examining the pulse, for we desire then not only to test the strength of the blood stream passing through the artery, but also the resistance the vessel itself opposes to that stream, by its greater or lesser degree of firmness, and the condition of the walls of the vessel. The examiner who sets himself to feel the pulse in a really useful way to himself and his patient bears the following points in his mind in doing so: he seeks to observe how many times the pulse-wave occurs in a minute—that is, he ascertains its frequency—he notes the rhythm of the process, and finds out whether each beat follows the preceding one in a regular sequence of time, or whether there is an irregularity in this respect; he gauges the volume of the wave and its power of resistance to obstruction by his finger, in this way becoming acquainted with the state of tension or of relaxation of the vessel-walls; and he runs his finger up and down the artery to feel if it has soft coats or the reverse. Thus, you see, the proper examination of the pulse is not the simple and routine affair which it generally appears to be.

### Nursing in North Germany.

BY AN ENGLISH NURSE.

A CORRESPONDENT writes:—

The best Christmas present I received last year was the promise from a friend to send me THE HOSPITAL and NURSING MIRROR every week. A little while ago, however, I read in one of the numbers that there are no women nurses in the military hospitals in Germany; that, and reading the letters from so many nurses abroad, makes me think you too may be interested to hear about nursing in this part of the world. In 1895 I came over here to learn German: in 1897 I tried to get into an English hospital, but, being too young, I was not accepted. Then I wrote to the "Red Cross Nursing Society" here, and was accepted, entering the hospital in October; and since then I have always belonged to the "Red Cross" nurses.

### THE GARRISON LAZARETH.

But at present I want to tell you about the military hospital, which is called "Garrison Lazareth." In winter there was so much influenza and inflammation of the lungs that I had to help at the "Garrison Lazareth," as the nurses too were ill. We have two nurses there, one in charge of the medical and one for the surgical wards. We are all called "Schwestern," even the probationers: we get our badge after three years' service, so there are no distinctions, as sister, charge nurse, &c.

### THE SCHÜLER.

As you know, conscription is in full force in Germany. If the soldiers prefer to join the army medical corps they need only serve at the front, as they call living in barracks, &c., one year; and if they have behaved well, they volunteer again for nursing, and are generally accepted and sent to the military hospitals, whence they are henceforth called Schüler (pupils), and wear their old regimental uniforms, except their forage caps, which have a different colour, and the helmets different plumes. . The Schüler have to attend classes and lectures, and pass two examinations; then they are free to leave the army, or become non.-coms., &c.; if they fail to pass both examinations they must remain six months longer, as a rule.

### THE SCHWESTERN.

Our work is to see that the sergeants and orderlies carry out the doctors' orders, and in very severe times, as, for instance, last winter, we take the patient's temperature, give the medicine, but specially see that the diet charts are properly attended to, and that the medical patients are well cared for, which requires some tact. The sergeants, orderlies, Schüler, and attendants, are particular about their titles, rank and rights, so it is not quite easy giving them orders, and far worse if one has to find fault. The doctors make their rounds before 10 A.M., and again between 4 and 5 P.M. Of course, in the surgical wards it is less difficult for us: we have the operating room—I really can't call it a theatre—entirely in our charge, but little to do with the patients unless there is something specially serious.

### HOURS ON DUTY.

We also have our certain hours on duty in the rooms where the serious cases lie—for instance, every day from 12 till 5 P.M., and, besides that, three nights a week from 9 P.M. till 2 A.M. during the winter. And often in the summer, from July till September, there is very little to do, and we seldom have duty, as the soldiers are generally sent to different places, and no fresh regiments arrive till autumn. Here no smoking is allowed at all, except in summer, and then in the garden, and those who are able always have to work hard. There are no flowers or pictures to make the large high rooms a little cheerful. Everything, to say the least, is very severe; but still the men seem contented enough, and we have a library and games. Our "Lazareth" is said to be well looked after; what the others are like I cannot imagine. Just lately all our nursing outfit has been looked at and improved.

### COMPULSORY SERVICE.

In the case of war we are bound to nurse the wounded. Also in epidemics we are bound to help nurse if the Government chooses to call us out. I am very fond of nursing naturally, and love my present work, parish nursing. But I must own I should never volunteer to work in a military hospital unless necessary. In the South of Germany there are lady nurses in one or two military hospitals, and in time there will be more, all over Germany probably. On the whole, I have had strange experiences during my two and a half years abroad. My desire to become a nurse at once, and not to wait five years, made me come here, but I am very glad now I came, though my chief aim is to be a Jubilee nurse in my own country, for there is no place like dear old England, and no sovereign like our beloved Queen.

### Nursing the Plague at Glasgow.

BY THE MATRON OF BELVIDERE HOSPITAL.

ALTHOUGH we had been prepared to expect plague for the last three years, yet it came upon us by surprise, and was in our wards before we knew that it had reached our shores.

From the bad character of our new arrival, I should not have been surprised if it had created some little apprehension amongst our staff. But, if it did, no one whispered such a thing, and there was a ready response for volunteers to nurse in the plague wards.

### PROTECTING THE NURSES.

The nurses selected were protected by having Yersine's serum injected. The result was very considerable discomfort or some time from backache and painful joints. They use 1-500 perchloride of mercury for washing the hands after attending patients. With these and other precautions we have little fear for our nursing staff.

The chief points in nursing plague are perfect cleanliness, free ventilation, and light nourishing food. In acute cases stimulants are pretty freely given.

### THE CASES.

Of the 27 cases we have had, some were so mild that they would never probably have been recognised had they not been under observation through being in contact with more severe cases. Some, again, had bubo in the axilla or groin, or both. Others had general glandular swelling.

Patients in the acute stage are very prostrate, often drowsy and stupid, but quite intelligent when roused. In some cases there is delirium. The buboes are very painful, but fomentations give relief; in some cases they absorb, in others suppurate and slough. The discharge is very offensive and infective.

### GENERAL PRECAUTIONS.

To prevent infection being carried, all dirty dressings are burnt, all linen and clothing used by patients are steeped in 1-500 perchloride of mercury, then put in the sterilizer and boiled for two hours. Formalin is used for blankets. All excreta and urine have perchloride added, and are then put in the autoclave and thoroughly sterilized by steam.

Over-alls are worn by the nurses while on duty.

### A BABY BORN IN THE PLAGUE WARD.

We had a fine baby girl born in our plague ward. The mother was admitted at night (a very bad case). She gave birth to the infant next morning, and died the following day. The infant seemed all right for a time, but swellings were seen in the neck, and death resulted on the tenth day. Our death rate has been very low as compared with other countries, and our great hope is that we may soon be rid of so unwelcome a visitor.

### Visiting Nurses for the Middle Classes.

### A CHAT WITH THE PIONEER OF DISTRICT NURSING.

By a Special Correspondent.

The question of nursing for the middle classes is exciting increasing interest. There is no difficulty in providing for the requirements of either the very rich or the very poor. People to whom money is no consideration can always obtain the services of the best trained nurses, and for the very poor there are the hospitals and the "servants of the sick poor," as Mrs. Dacre Craven nearly twenty years ago described the nurses who care for the sick in a room where it would be impossible for anyone to sleep who was not a member of the family. And because Mrs. Dacre Craven, more generally known still in the nursing world by her maiden name of Florence Lees, was the pioneer of district nursing, I sought an interview with her at the rectory of St. Andrew's, Holborn, in order to hear what she had to say on the subject.

"How can the need be met?" I inquired.

"To some extent it can be met by means of visiting nurses. They are particularly wanted for the middle classes.

### Personal Experiences.

"I have personally experienced the need of them. Some years ago when my husband was so ill I had two nurses, whom I boarded. But I could not put them up, and if I had been able to do so I should have needed an extra servant. They slept at their institution. I mention this as a case in point of the inconvenience of having nurses in the house. My experience is, of course, far from being unusual. I knew a person who was suffering from cancer, but there was no room for the nurse in the house. Eventually, one was obtained from the private institute attached to the Middlesex Hospital. She stayed with the patient all day, and went back to the institute at night. But cases are constantly occurring of professional people being unable to have a nurse in the house because they have not sufficient accommodation."

### The Question of Fees.

"Then there is the question of absolute inability to pay large fees."

"Yes, there is an immense gap between those who can pay two guineas a week and those who pay nothing. But there are nurses who are satisfied if they receive from 7s. to 15s. a week for visiting patients providing they get a sufficient number of cases."

"Do you think that there is a desire for visiting nurses among people who can only afford to pay small fees?"

### The Desire to Pay.

"I am sure that there is a general wish; it extends even to those who have been regarded as unable to pay at all. I remember the first case of payment being offered to a nurse of the Metropolitan Association. The wife of an ostler had been in the habit of paying sixpence a week to a little girl who "tidied up" for them, and when one of our nurses went to her she offered her the same amount. She said it was all she could afford, but she would rather pay it, and she did. I have known several cases of the wives of artisans insisting upon paying from 6d. to 1s. 6d. a week, and of others sending from 5s. to 10s. to us when the patient recovered. Last year the amount contributed to the Association by the poor—not paupers, but little tradesmen and artisans—was £7 18s. 6d."

"I proposed," continued Mrs. Craven, "that we should have a regular payment, but the committee would not agree, and one member said he was willing to give his time for the benefit of the poor, but not for that of the middle classes. A lady who subscribed to the Association wanted a nurse, but they would not let ~~her have~~ one until I made an arrangement for her. Occasionally, if district nurses can be spared from the poor to visit the middle classes it is an advantage."

"Is there a danger of the interests clashing?"

### Miss Nightingale's Opinion.

"I once asked Miss Nightingale if both interests could not be served, and I well recollect her reply. She said, 'You try it, my dear, and you will find that the poor will go to the wall.' The fact is that a different class of nurse is usually required to visit sick poor and the middle classes. Nurses who have a vocation for working among the poor should continue to minister to them. Such women esteem it an honour to cheer up the wretched and the dying. The best nursing in the world can only make the passage to death a little easier. But among the poor there are very rarely 'good cases' in which the born nurse rejoices, and I think that those who have not an intense love for ministering to helping sufferers naturally prefer 'typhoids' and 'operations' to 'old chronics.' One practical objection to the nurses to the sick poor also visiting the middle classes is the different conditions under which the two live. A nurse who had been tending a patient in the slums could not go in the same clothes to a middle-class house."

### Private Nurses Suitable.

"Then you think that it is, as a rule, better for the same nurse not to attempt to attend on both classes?"

"Yes, that is my opinion. Middle-class nursing is more attractive to the private nurse and more suitable for her. But some attempts to cater for the middle classes have been successful. A lady started a home at Hampstead with three nurses, her idea being that they should visit the middle classes and richer patients, or such as could pay a guinea a week and upwards. I should like to see the private nursing institutions, whether attached to hospitals or otherwise, reduce their fees to people who cannot afford to pay more than a certain sum."

"It would be necessary to know all about their circumstances?"

### Nursing a Vocation.

"Unquestionably. That would be a matter for the committee to inquire into. In some cases the fee might be remitted altogether; in others, reduced to half."

"Would not this tend to reduce the earnings of nurses?"

"The committee would have to regulate things. I may say that I deprecate the idea of women being trained to nurse solely for the purpose of making money, and I fear that many women who enter the profession have no vocation for it. Those who have not a special love for the work are never really successful. The difference between conscientious and other nursing is that those whose hearts are in their work will go anywhere, live anywhere, and do anything."

### The Training.

"Of course, you would have the visiting nurses to the middle classes fully trained?"

"By all means, just the same as nurses for the poor. The Metropolitan Association do not take any who have had less than two years' hospital training, and then they train them in district nursing for six months. But they ought to have three years' training."

"What training was considered sufficient when you commenced nursing?"

"For many hospitals, from one to three months. When Miss Nightingale started the Nightingale Home at St. Thomas's, she fixed the training at twelve months."

"You are, I believe, the oldest lady probationer now living?"

The "Lady Probationer."

"I was the second lady who applied for admission, and the only lady in the institution at the time of my training. That was in 1861, when it was not the fashion to nurse. When finally admitted (it was in 1865, as my family considered me too young in 1861), Mrs. Wardroper introduced me to the Sister as 'the lady probationer,' who was not to do the same rough work as the ordinary probationer, but I told the latter that I wanted to be an 'ordinary probationer,' and she rejoined that if I desired to become a nurse that was the only way to do it."

"How long did you continue nursing?"

"Until shortly after I was married. I worked as a probationer in the Deaconess Institutions at Dresden and at Kaiserswerth-am-Rhein. Then, after visiting all the chief hospitals abroad, I took charge of the male accident and female surgical wards at King's College Hospital. After that I secured further training and experience in the Hôtel Dieu, Enfant Jesus, &c., and the great military hospitals of Paris, subsequently working under the trained Roman Catholic district nurses of the poor in France."

Nursing the Sick and Wounded.

"That was before the Franco-German war?"

"Yes! During the war I volunteered to nurse the sick and wounded, and was placed in charge of the 2nd Fever Station of the 10th Army Corps at Marangue, before Metz. When the siege was over, I accepted an invitation from the present Empress Frederick to superintend her Royal Reserve Lazareth for wounded soldiers at Homburg."

"When did you commence interesting yourself in district nursing at home?"

The Beginning of District Nursing.

"In 1874, when, in conjunction with Lady Strangfield, I became honorary secretary of a sub-committee, with a view to the promotion, by the Association of St. John of Jerusalem, of an association for providing trained nurses for the sick poor in their own homes. In this capacity I visited most of the chief nursing institutions for the sick poor already existing in England, and drew up a report of their work and organisation. At the special request of Miss Nightingale I accepted the post of superintendent-general of what has since been called the Metropolitan and National Nursing Association, and it was a great gratification to me when the trustees of the Queen's Jubilee Fund decided to employ the association to train district nurses for the Queen Victoria Jubilee Institute."

### The Heaven-Born Matron.

She was a born leader. You realised it the very first morning that you saw her stand up in the great nurses' dining-room, and say grace before breakfast. There was a ring in her voice which told you she was meant to be a ruler. Yet there was nothing of the disagreeable autocrat about her. She did not give you the impression of ruling with a rod of iron, but with reins held firmly in a hand that understood its work. Hers was pre-eminently a strong character. She could no more have held the reins of government loosely, or dropped them from her fingers, than she could have done any other weak action, or altered that strong face of hers. In repose the face was stern in its outlines, but there was a rare charm—even fascination—in her smile, and her grey eyes, that looked straight at you, were full of kindliness. I have used the word "straight." It applied to her in every way. She was absolutely, entirely "straight" in all her dealings. You always knew just exactly where you were with her. If you were an evil-doer you might rightly dread her frown and short severe word of reproof. But if your conscience was clear you knew that you might invariably expect her kindly smile and cheery word.

She was always the same. No one feared that she would one day be all smiles and graciousness, and the next, if not "like Niobe, all tears," at any rate all frowns! Her temperament was perfectly equable—if it had not originally been so by nature, she had learnt to make it so. She was a self-controlled woman. None of her subordinates ever saw her in a passion, though she could be righteously angry. She never lost her temper. She never nagged or stormed; she was that very *rara avis*—a just woman. You instinctively felt that if things went wrong with you, she would listen dispassionately and calmly to every side of the question, and not give judgment hastily. Somehow you did not mind her severity—and she could be severe at times—because you knew she was always just.

Yet, though she was a strict disciplinarian, she could be most gentle and sympathetic. People went to her with their troubles and difficulties, because they felt she was so reliable and helpful; she would always put her whole mind into what you were saying to her. She seemed to grasp your situation, and enter into it, and her advice was very sound and wholesome.

There was a splendid unswerving rectitude about her which no one could help admiring. She never attempted to ingratiate herself with those set over her. She had infinite tact, and if she and her committee differed she set to work to explain wisely her point of view, and I believe, thanks to her tact, she usually ended by making it theirs as well! But she never "toadied" to them, or to anyone. If she thought a thing was right, she worked unceasingly to get that thing done, at whatever cost, and her influence over the whole hospital was wonderful. Well! she simply raised the tone of the place. We had dropped into slipshod ways, and the spirit of the hospital was not what it should have been when she came to us. There was a great deal of underhand conduct, and doing things behind the matron's back which you would not do before her face. Now it was curious that she never *said* much about this—she never did about anything—but a change crept over the whole Institution after she had been there a little while. The example of a perfectly upright woman made you ashamed of being anything but upright. It seemed impossible to try to deceive her, because she was so honourable herself.

She had a great power of inspiring personal loyalty and enthusiasm for the work—again, not so much from anything she said as by her own strong, enthusiastic personality. And she had the gift of observing us all, and of noticing our individual work, which made us especially anxious to work well. She was marvellously tolerant of all our failings and shortcomings—tolerant in the best and widest sense of the word, but never lax in discipline.

She was a broad-minded woman. Her rules were few and laid down on broad, simple lines, but they were enforced absolutely. There was nothing small or petty about her or her rules. She was that very beautiful personality, a single-minded, simple-hearted woman, and you could not think of meanness and of her in the same moment. In remembering her I think of those great words:—

> "Self reverence, self knowledge, self control,
> These three alone lead life to sovereign power.
> Yet not for power (power of herself
> Would come uncalled for) but to live by law,
> Acting the law we live by without fear;
> And because right is right, to follow right
> Were wisdom, in the scorn of consequence."

### The Nurse in Hot Climates.

By EDWARD HENDERSON, M.D., F.R.C.S. Edin., late Surgeon, General Hospital, Shanghai.

THE attention given of late years in England to the study of disease as manifested in hot climates renders it unnecessary for me to offer any apology for making the duties of the nurse in regard to these maladies a subject for separate discussion. Doubtless the trained nurse who has gained her experience in the wide field of general practice provided by a large hospital in England is already very fully instructed in all essential matters relating to the care of her cases, and if called on to exercise her profession abroad would soon, and on most occasions of her own initiative, make those modifications in practice which constitute the difference between nursing in London and Calcutta. But even she may find an advantage in hearing the more important points formulated beforehand, and learning something from the experience of others regarding the difficulties to be encountered, and the means by which these may best be overcome.

*Age.*—The age of the nurse herself is of importance; for it is a matter of common experience that such a radical change of the conditions of life as residence in the East implies is less well borne by very young women and by women who have passed middle life. As a general rule, the nurse who goes abroad for the first time should be not under twenty-five, and not over thirty-six, years of age.

*Medical examination.* — Examination by a competent medical man as to health-fitness is usually made a condition of the nurse's engagement, and even when this is not done the precaution is one which should never be neglected. In regard to such examinations, it may be stated generally that any departure from health which would lead to rejection or delayed acceptance of a life proposed for insurance on ordinary terms would constitute unfitness in a nurse for work in the tropics; and it is manifestly in the interest of the nurse herself that the discovery of any condition of the kind should be made early. A delicate physique, especially when associated with what is commonly spoken of as defective circulation—the hands and feet being habitually cold—is an unfavourable condition; and so too is a history of recurrent catarrhal affections of the intestinal mucous membrane: though perhaps neither of these constitutes positive unfitness.

*Protection by Prophylactic Inoculation.*—At the present date, there are four acute diseases of a serious kind to which the nurse who goes to the East may at any time be specially exposed, but against which, it is believed, a more or less lasting and effective protection can be obtained, either before she leaves England or when she reaches her destination abroad. These diseases are smallpox, enteric fever, cholera, and plague.

Against smallpox, a thorough primary vaccination done in infancy, if repeated once successfully in adult life, is regarded by many as constituting a sufficient protection. Since, however, the immediate result of vaccination is trifling, and the operation itself practically free from risk, its repetition every six or seven years is commonly practised. In the case of the nurse going abroad, it is better always to repeat vaccination when two years have elapsed since the date of its last performance, and to revaccinate in every case in which there is reason to believe that the last operation has failed. Although the addition of glycerin and improved methods of preparation have greatly helped to preserve the activity of vaccine, it would seem that its deterioration still takes place more quickly in a hot than in a temperate climate. On this account, and because of the immediate risk incurred when the nurse is brought in contact with natives on board ship and at the various ports of call, it is advisable that she should be vaccinated before she leaves England.

Protection against typhoid fever is sought to be established by the inoculation of an antityphoid "vaccine," a practice inaugurated at Netley in July 1896. Nearly 2,000 of these inoculations were done among the soldiers of English regiments quartered in India, during a period of twelve months, beginning in December 1898; and the results obtained were regarded as showing that if the practice were extended over the whole British Army in India, or the susceptible part of it, it would mean an annual saving of 1,000 cases, and nearly 200 lives. Professor Wright, of Netley, in commenting lately on the official returns of the outbreak of typhoid in the garrison of Ladysmith during the siege, expresses the opinion that it is at present impossible to determine precisely to what extent the inoculated were protected by inoculation, but at the same time speaks of the general results obtained as distinctly encouraging. The operation is one rarely attended by any really serious consequences, but it needs, of course, care in its performance, and usually entails on the patient a short confinement to the house, or even to bed.

Protection against cholera and plague is got by the prophylactic inoculations introduced by Dr. Haffkine, inoculations which have been apparently used with considerable success in India: in cholera, greatly reducing the number of the cases, though not affecting case-mortality; in plague, reducing the number of the cases, and lowering the death rate by more than a half. The nurse may on reaching her destination decide to make a trial of one or other of these two last prophylactics, but the operation is not one which is at all likely, in the present state of our knowledge, to be undertaken before she leaves England. The duration of the protection said to be obtained from prophylactic inoculations directed against typhoid, cholera, and plague has been variously stated: in the case of typhoid, it has been estimated at from eighteen months to two years; in cholera and plague, it is supposed to last during an epidemic, or a period of about six months.

*Outfit.*—On entering the tropics, the nurse must of course be provided with suitable clothing, but anything like a large outfit is a mistake. In most of the towns or districts in the East where European nurses are employed, there are peculiarities of climate, mode of life, and fashion, and the garments worn by the European inhabitants there are those naturally best suited to the varying conditions. Such clothing can generally be easily procured by the nurse on the spot on reaching her destination; if not, the conditions of modern transport will usually make it a simple matter for her to send to England for what she wants. She will, of course, leave behind her such details as to measurement, &c., as will enable her easily to communicate her wishes to the dealer. No one at the present date who travels on board an ordinary steamer carrying passengers need burden himself with anything like a medicine chest, but a small bottle of aperient pills will be often useful, and is easily carried. While admitting that the effect of "chill" as a cause of illness other than a simple catarrh is certainly much less than is generally supposed, it is still right to warn the inexperienced traveller that in the tropics she should provide against sudden changes of temperature by suitable alteration of clothing in a way she has perhaps scarcely been accustomed to do in England. When the change is one from heat to cold, and, as sometimes happens, occurs suddenly on board ship, she may have some difficulty in doing this; and she may here be reminded that, in the absence of thicker clothing, she can at once add materially to her comfort and safety by doubling the thin under-garments she is already wearing.

(*To be continued.*)

### Sketches from the Maratha Plague Hospital, Bombay.

By One of the Nurses.

The entrance is through a gateway of really imposing proportions. An expansive arch, crowned by little minarets thoroughly Oriental in appearance—artistic, too, when viewed from a distance; but a nearer inspection reveals the fact that the apparent blocks of granite are but bamboo mats, painted, in accordance with native taste, bright buff, and fitted into a light wooden frame of turquoise blue.

### The Patients.

A board that spans the arch states in English and Marathi that "This Hospital is for the special use of Mill-hands, Cartdrivers and Coolies." It has apparently achieved the object for which it was erected, for though some of the higher castes take advantage of it, the bulk of its patients are of the labouring class, and during the year 1899 three-fourths of those admitted came voluntarily for treatment. They have given it a name for themselves, and rather a pretty one. The hospital is situated just opposite the beautiful Victoria Gardens, and the natives call it the Ranicha Bág (or Gardens of the Queen) Hospital. The fame of it has spread far and wide among the Maratha community. I was first made aware of this fact by a poor delirious little boy, who begged of me to take him to the Ranicha Bág Hospital, for there he would get soda-water and all good things. I assured him that he was already there, and a cup of soda-water was held to his parched lips. "Not like that," he said, fretfully, "but bottles." Jealous for the reputation of our hospital, I showed him the newly-opened bottle and assured him that he should have as much as he wished for.

### The Wards.

On the left are five new pucca-built wards. The walls are of teakwood, the roofs neatly tiled, and the floors of concrete. The money for these beautiful buildings has been entirely subscribed by rich native gentlemen, and the work overlooked and supervised by a benevolent Mohamedan, the Sirdar, Mir Abdul Ali, Khan Bahadur, who for many months gave up every hour of his spare time for this good object.

At the end of the drive is a long row of wards, eight in number, built with bamboo posts and mats, whitewashed within and without. The floors are of mud, whitewashed too, and for cleanliness' sake frequently sprinkled with quicklime. Some of the wards hold twenty-six, and others from thirty to forty cots.

### The Epidemic of 1899.

During the epidemic of 1899, every cot held its burden of suffering humanity. One ward was reserved for women and children under observation, but all the rest were used for plague patients. Behind the first ward is a small shed built expressly for pneumonic cases (as this form of plague was supposed to be more highly infectious than the bubonic), holding six cots. Alas! not a single patient in that ward recovered, and when the cots were all occupied, the scene was almost too painful for description—the panting, gasping men were so patient and uncomplaining in their pain. Pneumonic patients seldom lose their consciousness, and this fact seems to make their condition even more pitiful than that of the other victims. Undoubtedly their sufferings are much more acute. Beyond the pneumonic ward is the shed containing the steam-bath apparatus. The steam bath was found at times to be a very useful adjunct, especially in temporarily allaying head symptoms and giving relief in cases of convulsions. In front of the wards stands the kitchen, and in front of the kitchen, during the busy time, might often be seen a quaint group of ayahs and ward boys making poultices. This business they carry out on a plan completely their own: squatted on the ground, they spread the poultices on the top of an old packing case with a rough piece of stick, and after much teaching they now manage to bring them into the wards scientifically folded and fairly hot. Lately, they have been supplied with poultice boards, but I feel tolerably sure that they will never be used unless the nurses are standing by at a table to spread the linseed. It would be far too great an exertion to be gone through unless under compulsion—and the poultices are numbered by hundreds.

### The Fly Nuisance.

Behind the kitchens are the tents set aside for the nurses. Breakfast and tea are sent down to us from our headquarters, and many a happy, though hasty, meal we have had in that little hut. The flies are the chief drawback to comfort; at one time of the year they were so numerous and so tiresome that we had to sprinkle sugar for them on the tablecloth before we ourselves could begin to eat. Fortunately, the flies appreciated our hospitality, and settled down, a black cloud, to enjoy the sugar. The same flies treat a fly paper with infinite scorn, and are a terrible trouble to the poor patients unless they happen to have some loving relation or friend in attendance who will keep the teasing insects off with light fans provided for the purpose.

### Lord Northcote and the Flies.

The hospital has been visited by Lord Northcote. His Excellency was much distressed on observing the fly worry, and gave a large sum of money, a portion of which he requested might be spent on punkahs to mitigate the annoyance. This kindly wish has now been carried out.

### Plague Epidemics and Prejudices.

During the first two plague epidemics we had no easy task in combating the prejudices. Many a time the patients have grasped our feet, praying us not to give the wet pack or stimulant mixture to their stricken relatives or friends: the icebags were continually removed, and the poultices too, if the patients objected to them. Now matters have changed for the better. The people have a quiet confidence in us as they realise that we work only for their good; and for some of our remedies, especially the nasal feed, they evince the greatest respect. The hypodermic syringe is still looked upon with great disfavour and suspicion, as many of the ignorant believe it to contain poison with which, they say, we kill the patients.

### Native Credulity.

One very common idea in the minds of these poor simple folk is that we may be persuaded to cure their friends if only they can offer us money enough. I well remember a poor woman taking me on one side, and, with an air of deep secrecy, untying a knot in her lugada (upper garment) and taking therefrom a two-anna piece and a pice, probably all she was worth, begging me to accept it and make her son well again. Another time two young men produced three rupees, which to people of their class must have represented quite a considerable sum, and besought me to cure their father, and give him *Chanagala Oushadha* (good medicine). Nothing, as I well knew, could save the dying man. I told them that their father was very sick, that we had already done all we could, and did not want their money; but their simplicity made my heart ache. It is not to be wondered at that people so ignorant and credulous fall an easy prey to those unprincipled persons who throughout successive plague epidemics have played upon the fears of their fellow-countrymen, and have lined their pockets with ill-gotten gain. A Hindu gentleman told me that during the few months he was house-surgeon of a large plague hospital in the native quarter of the city, he was offered numberless bribes, varying in value from 2 rupees to 500.

(*To be continued.*)

### Across the Seas.

### NURSING IN PERNAMBUCO, BRAZIL.

By A Correspondent.

Rather more than four years ago some of the English residents here decided upon starting a Nursing Institute having greatly felt the need of trained nurses during an epidemic of yellow fever in 1895. In January, 1896, two thoroughly trained English nurses were engaged to attend the English of this community, who number about two hundred. For the first three years the nurses attended patients only in their own homes, but since last September, in accordance with authority given at an extraordinary general meeting of the committee held in August, very suitable premises were secured at 5 Rua da Harmonia, known as the "Casa da Saude," formerly in possession of two Brazilian doctors.

### The Institute and Surroundings.

The house possesses all the facilities for a hospital on a small scale: the building stands in several acres of beautiful grounds, surrounded by flowers, fruit trees, palms, and lovely foliage. The climate is rather trying to newcomers, but on the whole it is really pleasant, the days being bright and fine, and we have always a breeze from the sea. Every evening, for about one hour, there is certainly a decided dampness in the air, more especially after the heavy rains. This year we have had a delightful rainy season; most of the rain has fallen during the nights, leaving the days clear and cool, with plenty of sunshine. The town looks very picturesque on a moonlight night, and reminds one of some parts of Venice.

Birds are a great feature here. It is very fascinating to watch the manœuvres of the humming-birds: they poise on the air, and dip their bills into every sweet flower within their reach. The mosquitos are very much in evidence; they plague new arrivals terribly. Although we are only eight degrees from the Equator, the health of the English residents may be considered good. Hence the nurses are not employed for many months during the year.

### The Cases.

The total number of cases attended by the nurses for the year 1896 was 17; 1897, 14; 1898, 11; 1899, 12. Since the hospital was opened last September nine have been treated as in-patients. Already the numbers have increased again this year, fifteen having been nursed up to present date, and there are still several more months before the end of the year.

### Patients from Ships.

The increase in the number this year is mainly due to the hospital being able to take patients from ships that are passing through. One patient so admitted is an American authoress. She was on her way to Rio, and suddenly developed a severe attack of facial erysipelas. She was put on shore here, and was delighted to find such a place as the Institute. She lost no time in gaining admittance, and appreciated everything that was done for her. In fact, she was a charming patient. Three sea captains have been treated as in-patients. One was such a queer old man—an American. He met with a bad accident some miles out at sea; when admitted he was suffering from hæmorrhage on the lungs and a fractured clavicle. He was most amusing. One of his remarks was, "Nurse, how did you and your pal *drift* out here? This is not the place for two such nice gals. Can't you get work in your own country?"

### Yellow Fever.

There have only been eleven cases of yellow fever treated by the nurses during the past four years. Seven recovered; four died. Miss May Clemetson, one of the nurses, died from same cause in the early part of last year. She contracted the disease while on duty, having only been in the country about two months. During that short time she made many friends, who felt that they had lost a splendid nurse and a good woman.

### No English Doctors.

There are no English doctors in Pernambuco. It would be a great boon to the English community if a good all-round practitioner started a practice in the town. All the doctors are Brazilians, and only one can speak a little English; hence, all directions and prescriptions are given in the Portuguese language, which is a great disadvantage to an English nurse until she can learn the Portuguese tongue.

### The Dom Pedro Hospital.

There is a fine building in the town, known as the Dom Pedro Hospital, for natives and foreigners of all nationalities. It is nursed by a Roman Catholic Sisterhood. The Rev. Mother very kindly allowed me to view the wards. The corridors and verandahs are very wide and lofty, the wards are large and well ventilated; most of the floors are polished wood, and the patients do the greater part of the polishing. They wear rubbers on their feet, and by moving them backwards and forwards manage to do it most effectively. There is one Sister in charge of each ward—thirty to thirty-five beds, which are nearly always filled. The majority of the patients are phthisical: a great number were suffering from skin diseases of various kinds, and I noticed several bad cases of beriberi. The patients keep all their worldly possessions, along with their food, under the beds, plates and other articles being side by side.

In every ward you find a very gaudy altar, the centrepiece, a statue, wonderfully decorated with tinsel gauzes and jewels.

---

### Life on a Navvy Settlement.

By a Trained Nurse.

At this time, when so many nurses are returning from South Africa, one cannot help wishing that some of them would turn their attention to the work of nursing the men on public works—i.e., navvy settlements, where large numbers of men are engaged (often in remote parts of the country) in making the docks, reservoirs, railways, &c., in which work probably the greater number of our militia and reserve men are employed in times of peace.

### The Kind of Work.

The life is full of interest, for even on those works where there are few serious accidents there are usually many small casualties in which first aid promptly and efficiently rendered materially assists recovery, and much pneumonia, acute rheumatism, &c., besides the too much despised chronic cases; and last, but not least, the minor ailments of the children, in which one's advice and treatment are generally gladly accepted. When once a friendly footing is established, much may be said on matters affecting the health and general well-being of the household. But even on the least dangerous of works a serious accident may occur at any time.

### A Serious Case.

One such case I remember well. A man's leg was so badly injured that immediate amputation was considered necessary. He had been hastily carried into the nearest hut, which happened to be much the dirtiest on the works—district nurses may imagine the kind of bed on which he was laid. A tourniquet was applied, and the patient allowed to rest until further help arrived, when, as much time had been already lost, the operation was at once performed, by the light of two tallow dips and a stable lantern, and without any of the usual preparations or paraphernalia. The patient was then placed on a stretcher laid across a cart, and conveyed to somewhat cleaner quarters four miles further on. He made a good recovery.

THE NEED OF PATIENCE AND HUMOUR.

Much patience is necessary, and a sense of humour not wasted, in this kind of work. One is tempted to make remarks more truthful than polite when sent for at 2 A.M. to see a middle-aged woman who had made herself violently ill by eating a quantity of plums, which, as she indignantly said, were "as hard as nails." Or one might be called out to find an exceedingly intoxicated "patient" sitting amongst the *débris* of his household goods, having finished a Saturday of frolic by smashing everything at hand and inflicting some small injuries on himself by the way. But whatever state the place or people might be in the nurse was not only perfectly safe, but was always listened to and treated with every possible mark of respect.

REFUSING TO ADMIT A BONE-SETTER.

Perhaps the most exciting time experienced at our little hospital was the morning on which we refused to admit a bone-setter, brought many miles at considerable expense by two of the most popular men on the works, to see a mate whose ankle had got a very ugly twist. "Not that we mean any offence to the doctor, who is a very nice gentleman, but two heads are better than one, *and this gentleman does nothing but bones*." They had not thought, and could not be made to see, that there was any reasonable objection to the "gentleman's" admission to the hospital in his professional capacity. They were bitterly disappointed, but went away without one angry word, and when next day they were offered the patient's discharge both he and his friends begged that he might be allowed to remain.

THE NEED OF NURSES.

When it is understood that on many public works there are from ten to twenty men lodging in each hut, and that all the arrangements are very primitive, it will be readily seen that, however well disposed his "landlady" may be, a man who falls ill with pneumonia, or any other acute disease, has but little chance of recovery in his lodgings. A woman who has a dozen men to cook and work for, and often several children of her own to tend, cannot be expected to spend much time or money on a man who may "slope" as soon as he has recovered, and his mates, though generally most unselfish and willing, can scarcely be described as skilled nurses.

THE NAVVIES.

It is often said that the illnesses and deaths of navvies are largely caused and accelerated by drink, and this is to some extent true; but is it not true also of other classes, not many of whom can plead the same extenuating circumstances? Their wandering lives, their laborious work, their lack of home comfort, all entitle them to our sympathy and forbearance. As in the army, the home influence is lost, and with it some of the home virtues, but more interesting many-sided work than that amongst them it would be difficult to find.

### A Word for "Special Nursing."

BY A SISTER.

HAVING been a hospital nurse for a good many years, I sometimes wonder if my own experience would prove useful to any of the noble army of willing martyrs who yearly offer themselves up on the hospital altars—which, by the way, can swallow an almost incredible amount of the disinterested zeal of these enthusiasts. If it were not for a touch of sadness in the thought, the number of young girls who ardently throw themselves into the nursing profession would almost be ludicrous. I think I may safely state that fifty per cent. of these are rejected after a short trial, owing, not infrequently, to their inability to grasp the fact that they have entered a profession to which they must devote their entire energy.

MISREPRESENTATION OF HOSPITAL NURSING.

Hospital nursing is not what it is so often depicted as by over-officious and utterly ignorant "visiting ladies," a romantic existence filled up by patting the ruffled pillows of the sufferers and giving them little bunches of flowers and pious booklets! It is, in reality, a life-long battle between health and cleanliness and the disease and dirt so prevalent amongst the necessitous poor; and an entire self-forgetfulness in the alleviation of the suffering of others. Consequently, those aspirants to the work who have idealised the common everyday work of the ward are speedily disenchanted and retire promptly from the field, to the great relief of their busy superior officers.

THE HEALTH QUESTION.

The class of nurse who has failed to whom I tender all my sympathy in her disappointment is the brave woman who faces the work with all her heart, and, putting all her strength into it, still fails. Why? Simply because her health cannot stand the demand put upon it. The long hours of standing and limited hours of rest tell at last, and a good, happy nurse breaks down and finds she must choose between her health and her work. Here my own experience comes in, and my advice to these willing workers, whose spirit is indeed willing if their flesh is weak, is to "pick up their strength, pack up their traps, and turn their attention to a less ambitious field."

TRY SPECIAL NURSING.

In other words, try working in the smaller special institutions—eye, ear, or throat hospitals offer an excellent chance of proficiency. Personally, I have nursed in ophthalmic hospitals for five years, and I believe I am a greater enthusiast about my work than I should have been in general nursing. It is a splendid profession, and so many women, who have failed to live at the high pressure which is inevitable amongst the staff attached to the large hospital infirmaries, give it up with a sigh of regret, because so few of them realise that they may still succeed in the special hospitals that flourish in provincial towns, in addition to those in London.

OPHTHALMIC WORK.

I think that in ophthalmic work especially there is great scope for the manifestation of extra proficiency of the nurse in charge. This has been proved to me over and over again by the gratifying comments of my patients, who can detect with marvellous rapidity the difference in touch of the fingers that have become expert in dressing eyes and in those of the new arrival, who, perchance, may come as "locum" from the general hospital, and to whom, as a rule, eye work is almost an unknown branch. Personally, I could never again work in anything else; but was it not Ruskin who truly wrote: "The constant duty of every man to his fellows is to ascertain his own powers and special gifts, and to strengthen them for the help of others?"

### Patients on the "Persia."

A TALK WITH ONE OF THE KILBURN SISTERS FROM LADYSMITH.

BY A CORRESPONDENT.

THE transport "Persia," with between three and four hundred men on board, has just arrived at Southampton, the Sisters in charge being Sisters Martha, Evangeline, and Clare, of the Kilburn Sisterhood, and Sister Grey, who joined the ship from Newcastle, South Africa. Thinking that perhaps the use of the word "Sister" in this case might be confusing, I asked Sister Martha to tell me a little about where and how she was trained as a nurse.

"I got my training," she said, "at our Accident Hospital at Rotherhithe, where we have a good many cases from the Southwark Docks. When I volunteered for Army nursing I was put under an Army Sister for a week at Maritzburg before I was given responsible ward work of my own. We had no cases of gunshot wounds there—the only wounds I saw were from soda-water bottles—all our men were suffering from enteric fever, dysentery, or rheumatism. On board we had some cases of influenza, too. We had chiefly to guard against relapses; one man had a temperature of 106 one day, but it went down the next. We left our really serious cases at Cape Town for Wynberg Hospital, I believe, so that they were only with us from Durban. As the 'Persia' is not a hospital ship, there was of course no convenience for serious cases, so we only brought convalescents."

"You lost two patients, did you not?" I asked.

"Yes, but no one expected they would recover; they had both had pneumonia, and death was caused by failure of the heart. We did all we possibly could to save them, but they died in the tropics within twenty-four hours of one another. We were so grieved to lose them. One of them had been a patient of ours at Ladysmith. They were both such charming men."

THE DAY'S WORK.

"What time did the day's work begin?" I inquired.

"At nine. We breakfasted at 8.30, which seemed very late to us. Then we went down and took temperatures, gave medicines, and sometimes did some of the dressings. Our orderlies were St. John Ambulance men, who were very efficient. There was not much to do during the afternoon, unless we were wanted for any special case. In the evening we went down again."

"Of whom did the medical staff consist?"

"The ship's doctor was P.M.O., and a R.A.M.C. doctor assisted. He was also lieutenant in one of the regiments."

"Had you any sick or wounded officers?"

"Captain Lonsdale was on board. He had been a prisoner in Pretoria. Two other officers had been wounded, but are now in good health."

"Was the food good?"

"Excellent. We had fresh meat every day, as there were oxen on board, and I believe one was killed almost daily.

RECREATIONS OF THE VOYAGE.

"Your work was not hard?"

"Oh, no; our chief aim was to keep the men amused during the voyage, and in this the officer in charge helped us all he could. We had a course of history lectures among other things, which Sister Evangeline gave."

"That was a novelty, was it not?"

"Yes, I think it was. I will tell you how it came about. One of our patients at Ladysmith was saying how anxious he was to get promotion, only he found the examinations such hard work, especially the history, as he had never been taught any at school. I told him it was a good opportunity to do some while he was in bed, and asked him a few questions forthwith. The men were delighted with the lectures. We had Bible classes, too, and we gave prizes for various things, such as drawing, essay-writing, and poetry."

"With what results?"

"Some of them did very well indeed. The officers all subscribed for the prizes, and we intended buying some things at Las Palmas, but when we arrived there it was too rough to land, so the prizes had to be given in money."

"Was the voyage rough on the whole?"

"No, not after the first, until we got into the Bay of Biscay. None of our men was sea-sick, fortunately."

THE RED CROSS SOCIETY.

"Did the Red Cross Society supply any comforts for the men?"

"Yes; I went to see Lady Furley at the Depôt at Cape Town, and she gave me everything I asked for, including playing-cards, dominoes, &c., to amuse the men on the voyage."

"What do you think of the British soldier as a patient?"

"He is a perfect saint when he is ill, so patient and grateful. Some of those who came over last time with our Sisters still come to see them every week; they love seeing the children in the Orphanage."

STILL AT LADYSMITH.

"Were you in Ladysmith during the siege?" I asked.

"No; the Sisters went up to Intombi a few days after the relief. We have left two Sisters at Ladysmith, and should not have returned now if we had not believed the war to be practically over. There are still about a hundred men there too ill to be moved; when they are gone, our Sisters will either return home or go on to India."

"One of the Kilburn Nursing Sisters died in South Africa?"

"Yes, Sister Teresa, who was at the Neutral Camp at Intombi. She was a great loss. I joined later, and was at Tin Town, about two miles from Ladysmith."

THE CATS AND DOGS.

"It was too funny to see the cats who had gone through the siege," Sister Martha continued. "They were so nervous at every sudden noise, poor things. There was a dog, too, who was a special favourite, and had been allowed to live—there was not enough food for all the animals—she was wounded in the fore-leg, and had had it amputated from the shoulder. She would let you stroke the place where the Mauser bullet was still embedded in her flesh. The strange thing was that her puppies all went about on three legs, holding up one paw! I thought it was so polite of them. The Tommies are devoted to pets. One afternoon we went for a walk past the camp, and there was such a noise going on that we asked one of the men if the dogs were always fighting. 'Morning, noon, and night,' he replied. Then we were told of a cat that was taken prisoner, with her owners, by the Boers; she would hide under the ambulance wagons while the firing went on, but she stood up to any other cat or dog, and fought valiantly with her front paws! Of course, Ladysmith had a cat called 'Emergency Rations.'"

### Presentations.

GUY'S NURSING INSTITUTION.—Miss Helen Wilson, who has been appointed Nurse Matron at the Prison Infirmary, Aylesbury, has been presented, on leaving, with a handsome travelling clock, from the senior members of the private nursing staff at Guy's.

### A Night in one of Her Majesty's Prisons.

By a Nurse.

We had all retired to our rooms on Saturday night, when there was a loud peal of the bell, and as a night summons is generally more or less exciting we were all on the alert to see what was wanted. I went down to answer the call, with Nurse D. whispering loudly on the stairs, "Be sure you ask 'Who is there?' before you open the door." Not being nervous, I disregarded her instructions and struggled with the door bolts in the dark. When the door was opened I found a stalwart man standing on the doorstep, who presented me with a note from the Governor of one of her Majesty's prisons. This was an urgent message to say that a baby had just been born in the prison, and requesting that a nurse might be sent to stay the night with the mother.

### Arrival at the Prison.

The warder waited whilst I dressed. And, as usual upon such occasions, every article of clothing turned itself inside out when it did happen to be found. However, I was dressed at last, and although the time seemed long I had been fairly quick, so with a deep sigh of regret for my cosy bed I sallied forth with my guide. It was a silent walk, and it seemed to me the warder was rather ashamed to be seen in the company of a nurse and a bag, which by the way he did not offer to carry for me.

We arrived at the prison at 11 p.m.: the warder rang, a voice said, "Who's there?" and we were let through a small door in the big gates by another tall man, who ushered me into a small room containing a desk, a chair, and a cupboard full of bunches of keys.

Before I was allowed to go any further my name was entered in a mighty ledger, and then, without a word, keys were handed by No. 2 to No. 1, and I was conducted through a gate into a large yard. Crossing this, we reached another door, where he rang the bell and waited.

### The Patient.

In a short time a woman appeared, wearing a brown stuff dress and a brown bonnet. I was handed over to this lady and the door locked behind me. Then followed a series of steps, passages, doors, corridors, all doors being unlocked and locked again, the keys jingling dismally in the midnight silence. It was just what I imagined a prison would be like. At last we reached the fourth storey, where we found the patient. She was not in a cell, but in the infirmary of the women's part of the prison--a ward containing two beds and a cradle, presumably for a baby. When I arrived another wardress was with the patient, and the two prepared to go at once and lock me in. However, I delayed for a little, wishing first to have some information and instruction about the patient.

### Locked In.

When I had learned all that was necessary, the two women left me, and there I was *locked in*, with ample opportunity of learning what a prison was like. The room was bare and dreary, with three windows of thick white glass, of course with iron bars outside. A fire had been lighted, but did not burn cheerfully, and the hard wooden chair provided by the Government for sick prisoners I did not find at all soothing—never had I realised before how many bones the human body contained. My patient was comparatively well, and I found the night very dreary, with nothing to break the silence but the chimes of a neighbouring clock. My thoughts were anything but pleasant when I remembered the other prisoners shut in cells all around me.

### The Patient's Crime.

At first I wondered why the patient did not ask for anything, but then I read the printed rules and found that as a prisoner she was not allowed to speak without being addressed first. The baby was quite fat and very like her mother: she slept peacefully, unconscious of the broad arrows stamped at intervals over the coarse clothing provided for such emergencies. Strange fate! that this infant should be born of a mother then undergoing a term of imprisonment for cruelty to her children! The mother seemed very hopeless and dreary, but I think she liked having me there.

Several times I looked longingly at the handle high up on the wall, which was the means of ringing a bell in the wardress' room, and very glad indeed I was to hear at last a bell ringing loudly at 6.30 a.m., which meant that the prisoners were to get up.

### Morning.

Just before 7 o'clock the wardress placed one eye at a tiny window in the door, and asked from outside if we were all right.

I felt a distinct longing to throw a piece of coal at the eye, for I was very tired and starved, having been at work all the day before, and having had nothing to eat since seven in the evening.

I cannot describe what a satisfaction it was to step outside the door, which I begged might be left open for a short time. The wardress was much amused at my gratitude for being let out, but then, as I said, it was all very well for her—she possessed the keys!

The cells of the other prisoners were unlocked one by one, and with three wardresses watching there was little chance of communication between them, although I did see one or two sly greetings as they passed each other on the stairs. One prisoner who came to my floor to fetch something slipped in to look at the baby, saying, "Ain't she a beauty!"

Even the prison cat seemed dejected, and wandered up and down the stairs, wailing loudly and apparently seeking a way of escape.

### A "Swill" Declined.

Presently, a wardress asked me if I would like to swill my face in her room, which kind offer I politely declined, saying I hoped to be in my own room soon.

What astonished me most was that the prisoners looked so cheerful, and indeed the wardress thought my distress for them quite unreasonable, and was inclined to be huffy about it, for she said: "Why some of 'em ain't ever known a 'ome until they come 'ere!"

After writing my report for the doctor, a message was sent to the governor saying I was ready to leave, and, the wardress having taken me through the many doors, passages, &c., we recrossed the yard, and at last I was in the open air, glad to know that my patient was doing well, and intensely relieved to have regained my freedom after a night in one of her Majesty's prisons.

### To Nurses.

We invite contributions from any of our readers, and shall be glad to pay for "Notes on News from the Nursing World," or for articles describing nursing experiences, or dealing with any nursing question from an original point of view. The minimum payment for contributions is 5s., but we welcome interesting contributions of a column, or a page, in length. It may be added that notices of entertainments, presentations, and deaths are not paid for, but of course, we are always glad to receive them. All rejected manuscripts are returned in due course, and all payments or manuscripts used are made as early as possible at the beginning of each quarter.

### The Neurotic Diathesis.

FROM A NURSING POINT OF VIEW.

By a Charge Nurse.

It is somewhat astonishing that in the present day, when so much is talked of the Science of Neurology, and so much more known than formerly, a more practical application of its teachings has not more commonly been brought to bear upon those lesser manifestations of nervous disorder with which a nurse is so frequently required to deal.

Is it not too often the case that a nurse who, although she may perhaps be able to run off glibly the name, function, and anatomical course of every nerve in the body, leaves hospital at the end of her term of training carrying with her very pronounced views on, and a huge contempt for, the hysterical and other neurotic conditions, under which descriptions she is prepared to place every incomprehensible symptom which she may observe, thus inviting, if she is a tactless woman, friction between herself and patient or the medical man in attendance on the case?

How frequently in hospital does one hear the many discomforts of which a patient complains disposed of lightly as being "only hysteria," a term fatal, as a rule, to intelligent sympathy. Nor is the nurse wholly without higher precedent in this simple method of disposing of a very tiresome matter. For instance, a house-surgeon going his late round, possibly after having fallen asleep in his chair and awakened tired and cross, hears from the night-nurse a trying report of unaccountable insomnia or inexplicable pain. 'Oh!" he growls, "Hysteria;" and the matter is instantly dismissed. And probably, when reporting to Sister in the morning, nurse receives the same assurance, "A most hysterical patient;" so that she is tempted in the future to diagnose this condition for herself, and consider a detailed report of it unnecessary.

Hysteria is most certainly a very trying disease—for we must not forget that it is a disease—trying as well for the patient as for the nurse. The symptoms are undoubtedly much more real to the patient than are those produced by a mere effort of imagination, and although the disease may be one in which firm treatment is desirable (as we are taught), it is also one in which discrimination should be employed, and on which a careful watch should be kept. This should be done as much to find the cause, if a definite one exists, as to prevent those tricks of deception which belong rather to the malingerer than, as is frequently credited, to the merely neurotic. For I venture to think that, in a certain number of cases, a cause may be found, where not perhaps suspected, in some uterine condition requiring surgical treatment. Hysteria in a patient admitted for some gynæcological treatment is assumed; but in a patient with some lesser ailment totally unconnected with the generative organs we naturally confine our attentions to the local condition and its possible complications, and ignore those organs which are presenting no more obvious abnormality than nervous derangement. Furthermore, seeing that hysteria may, and does, exist as one of the manifestations of the neurotic temperament without any diagnostic structural changes, an examination per vaginam under these circumstances, without further evidence, is quite unwarranted.

We know well that the congested condition of the uterus and ovaries immediately preceding the monthly flow produces a nervous tension influencing the moods of the average woman to an erratic degree. We have seen also how, after the operation of oophorectomy, the swelling of the tightly ligatured stump, and consequent pressure upon the ovarian nerves, frequently produces in the patient a nervous excitability bordering on insanity.

The histories of patients met with in out-patients' rooms and gynæcological wards tend to prove the assumption that many women suffer much in silence, either through ignorance, a false sense of delicacy, or through both these things together. The case of an old patient whom I nursed, a young married woman, who suffered from a bad prolapse uteri appearing through a completely ruptured perinæum—both conditions being of two years' duration—and who had neglected it, concluding that it must be one of the natural consequences of motherhood, which all women share alike, and feeling that it was too delicate a subject to discuss even with her own mother, is not an exceptional one, but illustrates the point. And this feeling is more common amongst the lower classes than is generally supposed, although existing chiefly with the unmarried. I need not point out that such a condition as the one mentioned above could not in any patient escape a nurse's observation; but in such instances as a minor prolapse, or one of the various other displacements, hyperplasia of uterus, split cervix, a form of endometritis unaccompanied by leucorrhœal discharge or other similar conditions, being present, it is quite possible that a little judicious sympathy and questioning may draw forth a statement of affairs for the surgeon's diagnosis.

An equivalent term of reproach is the word "neurotic;" yet who, having seen many of the poor nervous wrecks who present themselves for the Weir-Mitchell and other treatments, can withhold pity, even though guarding against any external manifestations of it. A neurotic person is one whose whole nervous system is hypersensitive and abnormally within his cognisance. The neurotic temperament is sometimes acquired, as during an exhausted condition of nervous system by overstrain, but more often it is hereditary. The family history of a neurotic whose tendency is inherited will show various functional complaints, as migraine, epilepsy, hysteria, asthma, with the visceral neuroses, as retention or incontinence of urine, constipation or diarrhœa, liver and stomach symptoms. Frequently, too, there exists the opium or alcoholic vice, the terrible craving itself a result of the general disorder; neuralgic affections are common amongst them, and, owing to the instability of their thermic centres, temperature charts with them have to be discounted—not from a deliberate plot on the patient's part to elevate the temperature, as I have sometimes heard asserted, but through the involuntary action of disordered nerves. They have, too, a morbid consciousness of the heart's action, feeling disturbances, as flutterings, throbbings, intermissions or faintness in its action: they also experience flushings and pallors of parts of the body's surface, and noises in the vessels about the head: their mental faculties are equally unevenly balanced, the subject having a great emotional capacity, often experiencing a season of mental exhilaration, closely followed by periods of intense depression, when every thought is clouded by blackness.

These conditions and their many variations are familiar to us all, and call for the best that is in us of tactful dealing and quiet kind firmness; for it is imperative that the patient should repose absolute faith in his nurse, being as open to receive impressions of strength and help as to imbibe the aches and pains of others near. Every endeavour should be made to draw the patient out of self, and encouragement should be given to the efforts of the will for mastery, lest, thrown back on self, an endeavour be made to deaden the misery of the senses by drugs or alcohol, and a life-long habit be thus formed. Surely this is the teaching that we should pass on to the nurses under our charge—"Absence of sympathy is not firmness, and indifference is not tact." It may be that in course of time it will be found desirable for nurses to spend six months in the wards of a lunatic asylum before training is considered complete, to gain practical experience in psychological work; but for the present we get our theory in the lecture room, and it remains with us to make practical application of it.

In nursing children one should remember that, although a child may be neurotic, its impulses are quite involuntary. A child's crying may be purely emotional, or may be caused by discomfort or pain—any cause is worthy of careful attention.

Another fact to be recollected as having a practical nursing application is that certain muscles are controlled by the will only by habit. A knowledge of this fact should instigate patience when nursing children of wet or dirty habits. From the bladder and rectum sensory filaments carry upwards the messages of unrest; the reflex circle, which should, by education, be under the control of the will, by force of long habit, which is not easily overcome, is free to respond and act spontaneously. Time and patience will accomplish much, but fear is a great weakener of the will.

The subject of idiosyncrasies, although not coming exactly under the above heading, is often confounded with it. Idiosyncrasy is a term denoting a peculiar temperament or habit of body, as, rice pudding will cause headache; dry toast will induce vomiting; opium will not induce sleep; astringents purge, and purgatives are a stringent. These things cannot be explained, but really exist, and must be taken into account in dealing with their possessors. Such statements from patients therefore should not be received with ridicule.

I offer these few suggestions to young nurses, knowing how easily they may be supplemented with a little thought. Some of us have learned our lesson of pity by witnessing in the post-mortem room the proofs of suffering which had been treated with scepticism as appearing quite unaccountable during life, but which, had it been recognised, would have obtained, if not relief, at least sympathy, the bestowal of which is one way of lightening another's burden. The lesson has been a stern one, but offers a moral for us all.

### Typical Patients.

### THE ADORED BABY.

During the holidays this year in a seaside village the baby who lives at the Court got very ill. As a rule, he was a beautiful, healthy, chubby boy, bubbling over with life and fun; now he seemed about to die. The doctor, knowing I had lately returned from a Small Hospital, came one evening to ask me to nurse the baby at night. He added, after my assent: "The mother is difficult to persuade; she won't let the child out of her arms, and she is overdone; you must see that she has rest."

I nodded, and accompanied him to the Court, he explaining meanwhile that probably the child would not live, for it was in great pain, and convulsive.

A rich stockbroker has a house next the Court, and his niece, a Big Hospital nurse, is staying there. She, being much interested in the case, was with the mother and baby when we arrived. The doctor's presence sent her downstairs to wait with me.

"Have you come to take the case at night?" she asked me, eyeing my uniform quizzingly.

"Yes," I returned; and added, for the sake of the look, "A Small Hospital."

"Oh," she said, disdainfully, "not a Big Hospital?"

The reputation of the Small Hospital was at stake.

"Of course, you know our hospital, and despise it?" I queried, meeting her scornful glance.

"Not despise," she answered, sheering round; "for I believe you get more practical work at your little hospitals." But she nodded her head, as much as to say, "We shall see;" and began: "It"—the baby—"has been put on Nestlé's milk; we don't believe in tinned stuff!"

"Neither do we," I returned; "but we are taught to obey orders implicitly."

"I consider the patient should be put to bed, with hot-water bottles: it is being carried about by its mother," she went on.

"The mother will keep the child warm. Perhaps it would break their hearts to separate them," I returned grimly.

"You must exercise your authority," she began, when the doctor beckoned me over the banisters. Half way up stairs, I looked back and smiled, being sorry for feeling irritated by a Big Hospital nurse; she was looking very undecidedly up at us, but she did not follow, and I did not see her again.

The baby is a darling—adored, spoilt and luxurious though he is. It is impossible not to shower love on him, while he merely takes it as a right, and so they all worship him. Now he would have none of the adoration from those around, but clung to his mother, pressing his bald white head into her neck, and hiding his face as if she were his only refuge, while she, poor soul, was holding him ever closer in her willing arms. The doctor, after giving some orders, left us. Presently the mother said, "Nurse, will you hold baby, if I give him up?"

'He would be better in the cot, I firmly believe, but, if you wish it, I will," and, holding out my arms, she put her treasure into them. The child began to moan, but she left him with a struggle. I followed the doctor's instructions as far as possible, with the child in my arms. His medicine made him cry, and that brought the mother back, frantic.

"Give me baby," she cried, seizing him; then rocked him wildly in her arms, calling him all the pet names she had ever known.

The baby's father, who followed her, began remonstrating and trying to soothe her, but, finding it was no use, sat watching her with his chin on his hand. A dear old lady, his mother, then came in great anguish offering help, and a frightened maid hovered near the door. The maid, delighted to do something for baby, fetched all the things wanted for the night, and we made the pretty befrilled white cot into a cosy nest, with hot-water bottles and baby's own downy pillows. At last, as the child grew quieter the mother's passion subsided; she was very tired, and passively let me take baby and lay him in the cot. As I covered him with the creamy eiderdown and rocked him the warmth and comfort of the nest sent him to sleep, so, going to the mother, I begged her to trust baby to me while she rested, and promised to call her if he were no better in the night. With a long look in my face and a quivering sigh, she held out her hand to her husband, and they went away.

All night I watched that tiny life ebbing and flowing on the borderland we do not understand. Once while tending the child he cried, and the mother, starting from her sleep, came like a white ghost and looked at him.

"He will want you—to-morrow," I said so she returned to bed.

When the night was nearly gone and everyone was deeply sleeping, and no sound broke the drowsy silence, the baby had a long, steady struggle for existence. Oh! what gives such weakly things that power of endurance and strength, when they can fight like any man without complaint? Then, worn out, he lay quite still, and I knew that he would live.

And in the morning the mother found her adored baby crowing and holding up his dimpled hands for her to kiss.

### Echoes from the Outside World.

### AN OPEN LETTER TO A HOSPITAL NURSE.

It was almost a foregone conclusion that Lord Roberts would succeed Lord Wolseley as Commander-in-Chief; but still it is pleasant to see in print that the highest military rank which can be bestowed upon a soldier of the Queen has been given to the hero of the hour. According to current gossip, his work, though of a totally different character, will be quite as arduous at the War Office as fighting Cronje or cutting his way to Pretoria. He has to advise the Government how to reorganise our land forces in England and its dependencies to the satisfaction alike of the Jingoes and of the Peace party, to do away with time-honoured traditions, and to introduce many things new. This, too, has to be carried on "in an atmosphere saturated with musty prejudice and inveterate devotion to red tape." But that gentle, pleasant manner of his, which wins the love of children as easily as the admiration and affection of soldiers, will go a long way towards preventing ructions, more especially as he has the immense advantage of having proved the success of his ideas in practice before he presents them in theory. It is hoped that Lord Roberts may take up his new office by Christmas. Meanwhile, the C.I.V.'s are on their way home, the Guards are starting shortly, and only the Gordon Highlanders are still pursued with the hard luck they have had all through the campaign. Some Boer ammunition being destroyed at Komati Poort exploded, and two of the famous Scotch regiment were, unhappily, killed, and a great number injured.

I have often grumbled at the enormities of laundries, where the washing is done by machinery, but I begin to think that the plague of torn clothes is preferable to the plague of Chinamen which is apparently coming upon us. You may have heard that we were threatened with a Chinese laundry; and now that it is an established fact, a few details may be interesting. A large establishment is being built at Hendon, where the men are ultimately to work; at present they are in a temporary laundry off Tottenham Court Road. According to the statement of one of the proprietors, large numbers of orders have been received from titled and wealthy people in Mayfair, Belgravia, and other parts of London, and it has become needful to send out circulars to intending customers to decline further orders for the present. A visitor to the temporary building states that clean-looking young Chinamen in clean clothes were seen washing at tubs, or at the tables ironing or goffering, and that the linen presented a most creditable appearance. The Celestials are not, nor will be, employed in packing or sorting, but simply in the harder work of washing by hand. This all sounds very delightful, but it is only admittedly the thin end of the wedge. A few isolated Chinamen in a London suburb can hardly be objected to; but if Mr. Benjamin fulfils his idea of "importing more and more Chinese, thousands of them, extending operations all over England and Wales, and even Ireland and Scotland, probably also going on to the Continent," it will become a very serious matter. Not only will the importation go a long way to deprive Englishwomen of work—that consideration would not, I know, weigh with those whose only desire is to buy in the cheapest market—but it will result in a Chinese quarter in every town. Travellers who have lived in lands where a large number of the yellow men are employed have painted the horrors of China town in such lurid colours that the notion of voluntarily arranging to repeat them sounds suicidal.

Many of you who saw "The Little Minister" when it was produced at The Haymarket, and probably look back upon it as one of the most enjoyable evenings you ever spent, will want to hear about Mr. Barrie's new piece at the Garrick Theatre. I fear to most of us "The Wedding Guest" will be a disappointment: not because passages of exquisite beauty and touches of quaint, delightful humour are lacking, nor because the characters, drawn with a realistic and vivid pen, fail to find clever interpreters at the hands of the various actors and actresses to whom they are entrusted, but because once more we have set before us that odious thing, a problem play. "The Wedding Guest," too, presents a problem which apparently even the author admits is too difficult to be possible of solution; for at the end of the last act the whole situation is left almost precisely in the same position as at the beginning of the first. The heroine, Margaret Fairbairn, is a young girl about to be married to Paul Digby. She is so innocent of the existence of evil that her innocence seems to have struck all her friends as her chief characteristic; and the first act, a pretty Scotch wedding scene, is refreshing and sweet, notwithstanding the unacknowledged presence of the "other woman" in the shape of "The Guest." In the second act Margaret Digby comes to the rooms where "Mrs. Ommaney," as she calls herself, lives with her baby; and the husband follows to look for her. The result is that Paul then thinks it his duty to tell his wife what he should have let her know long before, and she leaves him for her father's house. In the last act Margaret returns to her husband and offers to adopt the baby; but "Mrs. Ommaney," at least for the time, removes herself and her offspring from the scene of action. There the play ends, and notwithstanding some delightful scenes, notably the wedding rehearsal, the gambols of the child and the little Scotch nursemaid—cleverly played by a new actress, Miss Joan Burnett—and the quarrels of the old men over the game of draughts, one goes away sad and sorrowful. Nothing has been accomplished, nothing done; and even the remembrance of the tenderness of Miss Dorothea Baird, the strength of Miss Violet Vanbrugh, and the quiet force of Mr. H. B. Irving cannot remove the unsatisfactory impression left by the last production from the facile pen of the author of "A Window in Thrums."

The ways of the world are exceedingly wicked, we all know, but the methods of the female who, according to a Moscow paper, has lived by artificial mutilations seem to me to reveal depravity in a somewhat fresh light. This artiste—as she would, I suppose, describe herself—had been taught her trade by her husband, and after his death she stepped into his practice, which during the past two years she has successfully carried on, notwithstanding that the police have been on her track for some time. She is a Russian by birth, and her clients were mostly young men anxious to appear deformed in some way, so that they might be refused by the army medical examiner when called on to become a soldier. It is said that by injecting under the skin, close to the joints, some preparation of petroleum, she succeeded in producing a natural-looking contraction of the joint operated on. As her charge seems to have been a moderate one, her business increased, but it is not stated whether or not any permanent injury was produced by the injection. The woman was also much sought by those who knew that an injury could be turned to monetary account, and who requested her assistance for the purpose of defrauding the accident insurance companies. The last attempt, which led to the capture of the female mutilator, would have resulted, it was hoped, in a grand coup had it come off successfully. A young man was to arrange to fall out of a train, and the injury to the joint which would follow was to enable the victim to get compensation, not only from the railway company, but also from the accident insurance office. It was evidently another case of "the best-laid schemes of men and mice gang aft a-gley."

### Everybody's Opinion.

[Correspondence on all subjects is invited, but we cannot in any way be responsible for the opinions expressed by our correspondents. No communication can be entertained if the name and address of the correspondent is not given, as a guarantee of good faith but not necessarily for publication, or unless one side of the paper only is written on.]

### MADRAS GENERAL HOSPITAL.

"One Who was Trained There" writes: I was interested to read in the Nursing Mirror a letter from a "Resident in Madras," in which the writer wondered if the question of "pay" had anything to do with the difficulty of keeping nurses at the Madras General Hospital. No. I do not think it has: the difficulty lies entirely with the internal management, and until it is altered it is not probable that nurses will choose to remain.

"Another Who Knows the Hospital" writes: It is with much regret I see adverse criticism of the nursing arrangements in the Madras General Hospital in your paper. I assure you that Government have done much, and are well inclined to do more, the nursing staff having been increased to double the number, with an increase to more than double the pay, within the last few years. The nursing staff, with the exception of the matron and assistant-matron, who receive very good salaries, are entirely Eurasian, Anglo-Indian, or Native. The staff is still too small, but it is found that English nurses here expect more than can possibly be given them in pay, amusements, lack of all restriction, and a minimum of work: the right sort do not find their way out, as doubtless it is easy enough to get work in a better climate. In my opinion—I give it for what it may be worth—the organisation of the nursing department of this hospital is on an ideal basis, the management being entirely in the hands of medical officers who are responsible to Government. The matron is entirely subordinate to the senior medical officer, with the right of appeal to the Surgeon-General or to Government. I know for a fact that during her twelve years' service the matron's watchword, as far as nursing improvements are concerned, has been "Continually and consistently ask for more."

### THE SPARTAN NURSE IN PRIVATE WORK.

"One who looks on" writes: "I have been struck during the last few days by the aspect of the Spartan nurse in private work, and I have wondered whether it is really necessary for her to be so Spartan in her treatment of her patient." The lady who was being nursed spoke with warmth of the excellence of the nursing, "but," she said, "I wonder if nurse need be so severe. She does not seem to be at all sympathetic." Surely sympathy is compatible with good nursing! This nurse seemed to have no idea of looking after *small* comforts for her patient. The latter complained of backache; the nurse merely told her to lie still and go to sleep, instead of devising little ways of easing her—such as rolling a small drawsheet or cushion under her back, putting a pillow under her knees, or a hassock for her feet to press against. At bedtime the patient begged to have her hands and face washed. But no; the nurse ruled that she must go to sleep at once and not worry. Washing her face and hands would have helped to induce sleep, as a more experienced nurse would have known, whilst the avoidance of needlessly ruffling a patient's feelings is always wisdom. The patient begged to have her pillow shaken up—she was bidden to lie still! The nurse only left her training school (one of the largest in London) at Christmas, therefore her experience in private nursing was small; but is it not possible to teach nurses going into private work that this sort of needless Spartan severity towards patients is both foolish and wrong?

### THE NURSING ARRANGEMENTS IN SOUTH AFRICA.

"A Nurse in South Africa" writes: It has struck me that during the great agitation going on in England *re* hospital scandals in South Africa, a few words from one who has worked in two or three hospitals in various parts of South Africa for over six months might be of interest. First let me say that the sad state of affairs depicted by Mr. Burdett-Coutts was, as far as I can gather, peculiar to Bloemfontein. That such a state of affairs ought never to have existed there is obvious, but still it is extremely hard for arm-chair critics at home to understand the difficulties the authorities here have tried to contend with. However, what I wish to consider is the nursing arrangements. To begin with, a big blunder in the whole campaign is the fact that at the beginning of the war nurses were sent out in twos and threes, instead of in hundreds. Now the epidemic of enteric is just abating, and, let us hope, the big battles are over, nurses are flooding South Africa to such an extent one wonders where they will all be sent to. To me it seems that the whole system is at fault. Picture a hospital with from 500 to 700 beds, with enteric, surgical and medical divisions. At the beginning of the war perhaps there were nine to twelve nurses; now there are possibly twenty or more. This is what we see—an Army Superintending Sister who has to house keep, struggle with servants (frequently native, so many English maids have broken down out here), and also be always ready to give a cheerful, attentive ear to everyone from the P. M. O. downwards. Under her are a strange variety of women, from an old London trained hospital Sister with years of experience of ward management, down to perhaps a girl who has only just finished her training—she certainly may have a three years' certificate, but what experience can she have in managing perhaps 50 patients, to say nothing of orderlies, who are very quick to discover who is and who is not a ward manager, as well as a good nurse? Many of the Sisters out here are, I have no doubt, most excellent private nurses, or most useful in a place where they have someone over them, but they are no more capable of being placed in charge of a large number of patients than a junior teacher in a high school would be if she were suddenly promoted to head mistress. The case lies in a nutshell. The whole thing wants reorganising, like the War Office itself. The Army Superintending Sister cannot possibly go round each ward each day; therefore it is needful that capable, steady women, with experience of ward management or superintendence in a London or great provincial hospital, should be chosen and placed in charge of a division, or so many wards, and that they should be responsible that things are properly done, their standing being somewhat the same as a Night Sister at a large hospital.

As matters stand, it is more than trying to see the way things are worked, and how really capable women are able to do so little towards improving the condition of nursing out here. But a slight alteration in the system would make all the difference. Many hard things will be said of the Army Nursing Reserve Sisters at home, but there are very few who would not work their hardest for any patient when required, though, alas! one feels they too little realise that their work is sacred, and that a nurse's uniform and profession should be lived up to and respected as a priest's coat. If we could make each individual nurse understand her responsibility as regards her profession, then a great battle would be more than half won, instead of which a few foolish girls help to tar their fellow Sisters with the same brush as themselves, and make many a woman who loves and believes her profession to be one of the highest in the world feel wellnigh heartbroken with the hopelessness of trying to make the nursing profession respected.

APROPOS OF BATH-CHAIRS.

"I. DE V." writes: One day I was sitting as usual on a bench on the esplanade—watching the people passing and repassing—when a bath-chairman whom I had often seen dragging a very portly "fare" along, brought his conveyance to a full stop close by me, to take his breath and to wipe the "honest sweat" from his streaming forehead, having evidently just deposited his ponderous "fare" one at her destination. He was a tall, erect, spare old fellow, with a humorous Celtic face and a merry twinkle in his keen blue Irish eyes—an unmistakable ex-soldier. I was the amused auditor of the following soliloquy—addressed to himself! "Shure, it's meself that has been in the Cry-may-ah, an' I've fought the Rooshans an the Prooshans an' the Parley-voos—but" (here he again removed his battered old stove-pipe hat and vigorously mopped his venerable brow with a handkerchief that had seen better days), "but," he repeated—throwing the handkerchief indignantly into the hat and resuming his head-gear—"bedad, the hardest work iver I done was rowlin' ould weemin!" I felt that I could very heartily agree with the old Irishman's sentiments, for I had just left a chronic case where I had an old lady to "rowl," in consequence of which I am, like Paddy's old hat, very much the worse for wear! I shall certainly never undertake the like again; and I would fain warn my professional sisters who are about to take up permanent cases to stipulate that a donkey or a man (n-no, I do not mean to imply that the words are synonymous) be employed to draw the chair if one is in use. A woman is physically unfit for the work, and, added to the strain, anxiety and responsibility of nursing, it is just the "last straw," besides depriving some honest man of the job. There is only one excuse for doing it that holds good, i.e., if the patient is too poor to have help for the nurse. I merely give my experience in the matter because tired nurses, worn out with acute cases, seeking an easy one on their own account for a time—are sometimes induced to accept a post with a chair attached—and find too late that the hardest work they ever did was "rowlin' ould weemin!"

### The Nightingale Nurses.

THE Report of the Nightingale Fund for the year 1899 shows that fifty-three probationer nurses were admitted to the School at St. Thomas's Hospital in 1899; that twenty-one were discharged as unsuitable, or left from other causes; and that forty-four completed their probationary year's training.

The forty-four probationer nurses who completed their probationary year of training were placed on the register of Nightingale Nurses, and were taken on to the staff of St. Thomas's Hospital.

From the opening of the school in June, 1860, to the end of 1899, a total of 1,572 candidates have been admitted, and 943 have, after completing a year's probationary training, been placed on the register of Nightingale Nurses and received appointments in St. Thomas's or some other approved public hospital or infirmary, or District Nursing Institution for the benefit of the sick poor.

The annual gratuity of £2 allowed by the regulations for the term of three years to the nurses who have completed a year's service satisfactorily in some approved hospital or other institution for the benefit of the sick poor, was awarded to eighty-nine nurses, viz.:—Twenty-nine for their third year, twenty-nine for their second year, and thirty-one for their first year.

With reference to these gratuities, the Committee have decided that for candidates accepted for admission after October 1, 1900, these gratuities shall cease to be awarded, and they have under consideration the grant of a certificate of training upon conditions to be hereafter determined.

### Death in our Ranks.

ON September 27, at the Nurses' Home, Royal Infirmary, Edinburgh, there passed to her rest, writes a correspondent, one whose work for and connection with the Infirmary during a period of twelve years will be ever remembered with gratitude and affection. "Miss Annie Grant entered as a probationer towards the end of 1874, amid rougher surroundings and harder work than are the lot of the probationer of later years. At the end of her two years' training she went as Sister to the Thistlethwaite wards at St. Mary's, Paddington, where she worked for six and a half years. In 1883 she returned to the Infirmary as Home Sister. During the ten years she remained, her influence over the probationers was strong and for good. She had a keen sense of humour, and could laugh with her nurses as well as sympathise when trouble came. Her teaching was excellent, and many a frightened nurse on the eve of her first examination was cheered by her help and encouragement. Her old probationers, now scattered far and wide, have felt a lasting benefit from her teaching and example. Early in 1893 failing health obliged her to resign her post. After some months' rest, she was appointed Matron of the Royal Scottish Nurses' Institute, Queen Street, Edinburgh, which post she held till 1897. Obliged once more to give up work, she remained at home till called by the Royal Infirmary again in February 1900 to take the Matronship of the Home of Rest for the nurses at Colinton, a post for which she was peculiarly well suited, and which, unfortunately, she only held till August, when she found she could work no longer. She was removed to the Infirmary, and passed peacefully away in the Home where she had been so loved and valued, tended by one of her own probationers, and cared for as a sister by those she had worked with, and under the charge of the physician and surgeon she had known so long."

### Isleworth Infirmary.

SINCE July 1st, 1899, nine probationers have been promoted to be staff nurses at Isleworth Infirmary. Ten probationer candidates have entered the Training School. Of these one left at the end of the preliminary trial. There are now in the "Home" one probationer in her second year, and nine in their first. All the present staff nurses entered as probationers: one is in her fourth year, seven in their third, and six in their second year. Two nurses who gained their training certificates in the Infirmary have been promoted to be "sisters." The usual class and ward teaching has been given to the nurses and probationers by the Matron and ward sisters, and they have attended regularly the instructive lectures given by Dr. Fooks and Dr. Reynolds on Physiology and Anatomy. In addition Dr. Fooks has given several lectures on clinical subjects. The usual annual examination was held by Dr. Fooks and Dr. Reynolds in June, and the result showed that the nurses and probationers had worked well and profited by the careful instruction given them. Dr. Seymour Sharkey, of St. Thomas's Hospital, held the second final examination for nurses in their third year on July 1st and 5th, and five nurses passed successfully.

During the year all the sisters have received a course of instruction in massage from Miss Knight, certified masseuse. The Guardians having secured her as an instructress, the nurses and probationers will now receive regular instruction in massage. The Guardians are considering plans for enlarging the Nurses' Home.

### Travel Notes.

By Our Travelling Correspondent.

### LIX.—A WET NIGHT IN THE BLACK FOREST.

Few Continental travellers are strangers to the feeling of boredom, not to say desolation, which descends upon one when torrents of rain fall steadily upon a summer evening—rain that sternly extinguishes all hope of going out, even protected by macintoshes and umbrellas—and this feeling is intensified if you are a sojourner in a charming but primitive hostelry, where there is no sitting-room, save a meagre little square hall considerably open to the winds of heaven and to the hurried and noisy incursions of heated waitresses carrying away the displeasing remnants of a generous German repast.

Such was our melancholy position when our courteous host announced that a wandering troupe of Tyrolese singers and dancers proposed to favour us with a performance in the verandah.

The little company consisted of four men and two girls, mostly brothers and sisters. The youngest brother, who was also primo tenore, was the conductor, manager, and in every way the moving genius. I must tell you that wandering musicians from the Tyrol are totally different to the tramp-like people calling themselves strolling players in England. The Tyrolese musicians belong to a respectable class, and during the winter work in factories or in some branch of the timber trade. They are almost without exception really worth listening to as singers, and I believe go through a considerable amount of training in their art. I asked them questions on this subject, but owing to their patois and my own imperfect knowledge of German I am not sure that I thoroughly understood Resi, the prima donna.

The night we first saw them they had tramped 14 miles up a mountain road, carrying their kit with them, and proposed to tramp another ten the following day. They were a fine muscular set, with the exception of one of the women, who appeared of a feeble physique, but her voice was magnificent. The dress of the women consisted of black skirts, very full, with broad bands of blue round the bottom, large full aprons of vivid green, white chemisettes open at the neck, and with short sleeves and outside stays of black, across the front of which hung silver chains with medals and coins attached; some of the coins were very large, such as 5f. pieces. Fraulein Resi, who was a beautiful woman as well as a fine singer and dancer, was rich in these adornments. They numbered 15 on her massive and Juno-like figure. The whole costume was completed by a pink ribbon tied round the waist and falling over the green apron in long streamers, and a rather ugly flat black beaver hat with a yellow cord and tassels.

The men were no less picturesque. Thick boots, and stockings of vivid green—these came no higher than the calf of the leg. Black breeches, very short, left exposed the knees of the wearers; beautifully clean white shirts showed off marvellous braces of many hues. But their belts were a joy and wonder, and are heirlooms descending from generation to generation. The art of their manufacture has now disappeared: they were of stout black leather, and had broader pieces in front, in some cases as large as a cheese-plate. Each one had elaborate designs embroidered on the front, which I assumed to be worked in silver thread, but the Tenor took his off for me to examine, and to my astonishment I found the whole thing to be executed in minute portions of the small quills of birds. In winter they wear sumptuous waistcoats, with large emblems (mostly of a religious character) worked in the centre in gold thread. Their coats were grey faced with green.

They gave us most delightful part songs, with plentiful "Yodelling," accompanied by a zither and guitar. Most of these songs have more or less of dramatic action, either pathetic or comic.

But the feature of the performance was the "Schulplatel-Tanz" by Tobias and Resi. This is a most graceful and curious dance, descriptive of love and courtship, and is certainly one of the prettiest things I have ever seen; at the same time, the efforts made, especially on the part of the man, the vigorous leaps and bounds and gymnastic contortions generally, soon showed me the reason of his casting off all unnecessary garments. Towards the conclusion, when the swain's eloquence, soft looks, wily ways and muscular exertions, have begun to make way with the lady, they turn round and round each other in the most marvellous style, and he takes her in some mysterious way by the hands level with his shoulders, and twirls her round and round like a teetotum. The triumphant conclusion is reached when they rush forward, cross their arms together above their heads, and he salutes her with a resounding kiss! The girl told me, in answer to my compliments on the extreme grace of the dance, that they have done it together from the time that she was five and her brother seven. It seemed an exhausting affair, and no wonder Resi sipped delicately at lemonade and Tobias unblushingly mopped his forehead and sustained fainting nature with a glass of lager beer; but he told me that he did not venture on that indulgence often, because it was bad for the voice.

Resi confided much to us in answer to our appreciative remarks. She said they had old parents at home, and if their earnings fell off owing to bad weather the poor old people would suffer in the winter. She was made extremely happy by the present of a fat English half-crown to add to her adornments, and started off jubilant to tramp to Rippoldsau the next morning. I fancied I saw some tender glances bestowed on her by the zither player, an enormous giant, who seemed strangely placed before the humble little instrument, so I hope the Fraulein's future is happily arranged. The large man was decidedly in subjection, and gladly allowed himself to be laden like a pack-mule with her belongings. The younger brother was married, and was putting by all the money he earned on his wanderings to start five little Tyrolese of his own, in life.

I took photographs of the various members of the troupe, to their great delight, and look forward to the pleasure of sending them off next week.

### TRAVEL NOTES AND QUERIES.

Rules in Regard to Correspondence for this Section.—All questioners must use a pseudonym for publication, but the communication must also bear the writer's own name and address as well, which will be regarded as confidential. All such communications to be addressed "Travel Editor, 'Nursing Mirror,' 28 Southampton Street, Strand." No charge will be made for inserting and answering questions in the inquiry column, and all will be answered in rotation as space permits. If an answer by letter is required, a stamped and addressed envelope must be enclosed, together with 2s. 6d., which fee will be devoted to the objects of the "'Hospital' Convalescent Fund." Any inquiries reaching the office after Monday cannot be answered in "The Mirror" of the current week."

Outfit for the Cape (E. H.).—The clothes required would be much the same as for England, but the severe changes in morning and evening temperature necessitate woollen underclothes. Look for answers to your other questions under "Notes and Queries."

### Examination Questions for Nurses.

OPENING OF THE AUTUMN SESSION.

MOST of us have now returned from our holidays and begun to settle down to the usual business of life, and I hope that the pleasant scenes and refreshing breezes encountered on foreign and home travel have strengthened and invigorated all our nurses who stood so much in need of the stimulant of fresh scenes and bracing air.

Last year the competition for these examinations largely increased, and the standard of work was very satisfactory, a very large proportion of the answers being excellent. However, there is no such thing as standing still; either we progress or retrograde, so that I trust this session will show continued improvement, and, above all things, an increase in the employment of "common sense."

Oh! what a difficulty there appears to be in meeting with that rare gift! It would seem that no sooner does a nurse settle down to answer a simple question on a subject that presumably she is well acquainted with, than some malevolent imp instantly obscures her mental vision—common sense and due appreciation of relative values disappear in a mist, and instead of a plain answer to a plain question we have pages of poor stuff, wandering far afield on branch lines of twaddle.

I have frequently had occasion to complain of untidiness and carelessness in many of the papers, and I ask competitors to bear in mind that "what is worth doing at all is worth doing well." A nurse who will be guilty of a slovenly paper is more than likely to be also guilty of a slovenly ward, and those who are not faithful in little will certainly not be faithful in much.—THE EXAMINER.

QUESTION FOR OCTOBER.

In a case of inguinal or femoral hernia (in private work) what steps should you take to alleviate suffering pending the arrival of the doctor?

RULES.

THE competition is open to all. Answers must not exceed 500 words, and be written on one side of the paper only. The pseudonym, as well as the proper name and address, must be written on the *same* paper and *not on a separate sheet.* Papers may be sent in for fifteen days only from the day of the publication of the question. Failure to comply with these rules will disqualify the candidate for competition. Prizes will be awarded for the two best answers. Papers to be sent to "The Editor," with "Examination" written in the left-hand corner of the envelope.

### Autumn Novelties for Nurses.

BY OUR SHOPPING CORRESPONDENT.

GARROULDS'.

THE cooler days of a late but beautiful autumn remind us that it is time to think of warmer clothing suitable for the coming season, and we cannot do better than visit the well-known and deservedly popular establishment of Messrs. Garrould in the Edgware Road. Ever to the fore in variety and design, the intending purchaser will find something adapted to her needs, and at a price that brings it within the compass of the slenderest of purses. Nurses who, like other professional women, have to study economy, before selecting a gown are recommended to write for a selection of patterns: some of the new materials are very soft and beautiful and will amply repay the making up. The "Denrick" beige is a novelty to which we have quite lost our heart. It is soft and very light, and will make a charming dress for winter wear. It likewise possesses the merit of washing well. The selection of washing materials in cotton, calico, and linen has been largely increased, and some of the new shades are simply lovely. The "Melville" zephyrs are particularly fascinating. There are samples of both checks and stripes, and in the latter we particularly admired one in pale green, and another in heliotrope. There are some charming hades in plain design of the same material. Stripe drill and Halifax cloth are effective, and are bound to give satisfaction to the most critical of purchasers. The "Red Cross Catalogue" contains illustrations of all the newest designs in made-up uniform. Of cloaks there is an infinite variety, but we notice with regret that the old time-honoured and never-to-be-improved-on "circular" is conspicuous by its absence. In our opinion, it is the most refined and lady-like form of uniform cloak that was ever brought out, and nurses who study their appearance will prefer it to all others. There is a *cachet* about it that is possessed by no other cloak At the Woman's Exhibition at Earls Court Messrs. Garrould are showing in a case a wonderful set of models representing the uniform worn by nurses at the principal London Hospitals, and in a large case adjoining there is a selection of aprons, caps, wallets and other surgical appliances. There is also a section devoted to books on nursing subjects, where nurses may rest and have an opportunity of making themselves acquainted with all the most recent works.

EGERTON BURNETT.

THE samples we have just received from Egerton Burnett, Limited, Wellington, Somerset, show that this famous firm is in no danger of losing its reputation for all that is newest and most varied in fashion and material. The serges and tweeds are more fascinating than ever this season, and the choice is simply endless. The "Dartmoor" is a thick serviceable tweed, which would look equally charming made up either as a cycling suit or a cape or loose driving jacket. It is in several good shades, just relieved by a thin thread of a contrasting colour, forming a large check. Then there is the "Solway" coating, a stout cloth that will stand any amount of hard wear; while the varied shades of a beautiful fabric called the "Stirling" would lend itself admirably to the softer draperies suitable for home wear. Nurses would find it especially suitable for warm uniform wear, and the very reasonable price of 1s. 11½d. per yard, 49 inches wide, is one which will bring it within the reach of all. Another good material for stout country wear is the "Forfar," and we particularly admire a dark drab with a green check running through it. Scotch Wincey is a deservedly favourite material, and Egerton Burnett's samples are the prettiest we have seen for a long time. They will not only make charming dresses, but are equally adapted for underwear and nightdresses. To rheumatic people, or nurses who are apt to be called up at night, with the attendant risk of taking cold, we cannot too strongly recommend clothing made of this material. A soft flannel, "The Ladysmith," arrests our attention. What ideal blouses it would make! Intending purchasers will be interested to know that it is 32 inches wide and costs 1s. 4½d. a yard, and there is an especially sweet design in pale blue, with a narrow dark line running through in stripes. In washing materials the selection is more abundant than ever, and critical indeed must she be who cannot make a selection from the really beautiful examples before us. The drills, zephyrs, linens, and galateas are of the daintiest, and the shades are infinite. Dressmaking at reasonable prices is a speciality of this firm, and all the purchaser has to do after selecting her material is to send the measurements—instructions for which are enclosed with the patterns—and in a very few days she receives a costume made up in any style for which she has a preference. Cloakings are a line to which we call special attention, also the famous serge in various shades of navy and black. Patterns are sent post free to any address on receipt of a postcard giving name and address.

### Appointments.

ROYAL HANTS COUNTY HOSPITAL, WINCHESTER.—Miss A. A. Gwyn has been appointed Matron. She was trained at the Royal Hants County Hospital, and has since been in succession Matron at the General Hospital, Birmingham; the General Infirmary, Worcester; the Bath Hospital, Harrogate; and Lady Superintendent at the County Hospital, York.

LANCASTER UNION INFIRMARY.—Miss Agnes Bowen has been appointed Superintendent Nurse. She was trained at Birmingham Infirmary, and has since been Charge Nurse for three and a half years at the Chester Union Infirmary. She holds the L.O.S. certificate.

HOSPITAL OF S. FRANCIS, NEW KENT ROAD, S.E.—Miss Agnes Mary Boys has been appointed Matron. She was formerly Matron of Uckfield Cottage Hospital.

BABIES' CASTLE, HAWKHURST.—Miss H. Elliott has been appointed Matron Superintendent. She was trained at Addenbrook's Hospital, Cambridge, has held the post of "Sister" at the Hospital, Rotherham, and the General Hospital, Birmingham, and for the last two years has been Assistant Matron at the Infirmary, Isleworth.

THE INFIRMARY, ISLEWORTH.—Miss G. Rabarts has been appointed Assistant Matron. She was trained at St. Marylebone Infirmary, was three years Ward Sister at St. Saviour's Infirmary, Dulwich, and for the last four years has been Night Superintendent at the Infirmary, Isleworth.

H.M. FEMALE CONVICT PRISON INFIRMARY, AYLESBURY.—Miss Helen Wilson has been appointed Nurse Matron. She was trained at Guy's Hospital, where she was afterwards Staff Nurse, District Midwife (training pupils for the L.O.S.), and nurse on private staff. In addition to the three years' certificate, she holds the silver medal for five years' service from Guy's Hospital, and the L.O.S. certificate.

ISOLATION HOSPITAL, FINSBURY PARK.—Miss Ada J. Farr has been appointed Nurse Matron. She was trained at Monsall Hospital, Manchester, and has since been attached to nursing institutions in London, and Weston-super-Mare, Somerset.

### Minor Appointments.

EAST LONDON HOSPITAL FOR CHILDREN.—Miss Lucy Eastmead has been appointed Ward Sister, and Miss Jessie Smith has been appointed Out-patient Sister. Miss Eastmead was trained at the Children's Hospital, Liverpool, and the Hospital for Consumption, Brompton. She has since been night sister at the Victoria Hospital for Children, Chelsea. Miss Smith was trained at King's College Hospital, and has since been sister at the Royal Chest Hospital, City Road, and temporary sister at King's College Hospital.

KINGSTON UNION INFIRMARY.—Miss L. Parry has been appointed Charge Nurse. She was trained at Birmingham Infirmary.

WEYMOUTH ROYAL HOSPITAL.—Miss Miller has been appointed Staff Nurse. She was trained at Victoria Hospital, Folkestone, and has since been assistant nurse at Newark Hospital.

WALSALL AND DISTRICT HOSPITAL.—Miss M. O'Neill has been appointed Charge Nurse. She was trained at the Infirmary, Isleworth, where she has been four years.

HOMERTON INFIRMARY.—Miss Hannah Tucker has been appointed Charge Nurse. She was trained at the National Hospital for the Paralysed and Epileptic, Queen Square, where she has since been staff nurse. She has also been staff nurse at Vallance Road Infirmary, Whitechapel, and Charge Night Nurse at St. Pancras Infirmary.

### For Reading to the Sick.

THOU that makest the outgoings of the morning and evening to praise Thee, Thou visitest the earth, and blessest it.—*Ps.* lxv. 8, 9.

As on my bed at dawn I mused and prayed,
I saw my lattice prankt upon the wall,
The flaunting leaves and flitting birds withal—
A sunny phantom interlaced with shade;
"Thanks be to Heaven," in happy mood I said,
"What sweeter aid my matins could befall
Than this fair glory from the east hath made?
What holy sleights hath God, the Lord of all,
To bid us feel and see! we are not free
To say we see not, for the glory comes
Nightly and daily, like the flowing sea;
His lustre pierceth through the midnight glooms,
And at prime hours, behold! he follows me
With golden shadows to my secret rooms."
—*C. Tennyson Turner.*

**Reading.**

NATURE has two great revelations—that of use and that of beauty; and the first thing we observe about these two characteristics of her is that they are bound together, and tied to each other. The beauty of Nature is not, as it were, a fortunate accident, which can be separated from her use; . . . . beauty is just as much a part of Nature as the use, they are only different aspects of the self same facts . . . . all the colours of the landscape, the tints of the spring and autumn, the hues of twilight and the dawn, all that might seem the superfluities of Nature, are only her most necessary operations under another view; her ornament is but another aspect of her work, and in the very act of labouring as a machine she also sleeps as a picture. So in the sphere of space—the same hills which serve as the measure of distance to regulate all our motions also make the beauty of perspective.—*J. B. Mozley.*

Two souls there are in nature and in life—
The soul of Beauty and the soul of Truth—
Towards which we yearn and strain with reckless strife,
Along paths fraught with malice or with ruth;
In the red face of ridicule and scorn
Men sought, and still must seek for thee: for within
(In spite of all earth's sorrow and her sin)
The soul is to the search and manner born.
And still, in looking Beauty in the face,
With strong prophetic joy we recognise
Something of what we may be, as we trace
Our own dim shadow in her lustrous eyes;
Nor may we part such with a dull harsh rule—
Beauty is *true* and Truth is *beautiful!*

THERE is religion in everything around us, a calm and holy religion in the unbreathing things of Nature, which man would do well to imitate. It is a meek and blessed influence, stealing in, as it were, unawares upon the heart; it comes quickly, and without excitement; it has no terror, no gloom in its approaches; it does not rouse up the passions of man; it is fresh from the hands of its Author, glowing from the immediate presence of the Great Spirit which pervades and quickens it.—*Ruskin.*

A NEW day rose upon me. It was as if another sun had risen into the sky; the heavens were indescribably brighter, and the earth fairer; and that day has gone on brightening to the present hour. I have known the other joys of life, I suppose, as much as most men; I have known art and beauty, music and gladness; I have known friendship and love and family ties; but it is certain that till we see GOD in the world—GOD in the bright and boundless universe—we never know the highest joy. It is far more than if one were translated to a world a thousand times fairer than this; for that supreme and central Light of Infinite Love and Wisdom, shining over this world and all worlds, alone can show us how noble and beautiful, how fair and glorious they are.—*Orville Dewey.*

### Notes and Queries.

THE Editor is always willing to answer in this column, without any fee, all reasonable questions, as soon as possible.

But the following rules must be carefully observed:—

1. Every communication must be accompanied by the name and address of the writer.
2. The question must always bear upon nursing, directly or indirectly.

If an answer is required by letter a fee of half-a-crown must be enclosed with the note containing the inquiry.

*Books.*

(1) Can you recommend any books on (1) instruments, their names and uses, with illustrations (2) on theatre work and operations?—*B. O. X.*

1. The illustrated catalogues of the best instrument makers are exceedingly useful for the purpose. 2. Dr. Watson's "Handbook for Nurses" would most likely suit you.

*Children's Nurse.*

(2) I should be grateful if you would kindly inform me how and where I could best be trained as children's nurse?—*M. J. T.*

There are several institutions offering training in children's nursing. The Norland Institute, 29 Holland Park Avenue, London, W., is the pioneer, but if you mean by "a children's nurse" a nurse for sick children, you must be trained at a children's hopital.

*Institution.*

(3) Can you give me the names of any orphanages or homes where girls are sent out? Perhaps a fellow nurse could recommend a kind good home with salary for a girl with a knowledge of laundry work.—*Nurse Edith.*

The Girls' Friendly Society, 39 Victoria Street, S.W., is one of the largest societies for the protection and help of girls. Apply to the Secretary.

*Experienced Probationer.*

(4) I have had three and a-half years' fever training and now wish to become a probationer in a general hospital. I should like to know if all hospitals object to probationers who have been in other hospitals.—*Bertha.*

Most matrons object to probationers with elementary training from other schools. The case is different for one fully trained in a special branch.

*Lady Roberts' Nurses.*

(5) Will you kindly tell me anything you can of Lady Roberts' nurses. I should like to know to whom to apply, and if anyone is acting for her whilst she is in South Africa?—*Irish Nurse.*

Lady Roberts selects her nurses from personal knowledge. She never receives applications.

*L. O. S.*

(6) Would it be possible to work for the L. O. S. and to do the practical work as well in a month? Where can teaching be obtained most quickly and cheaply?—*F. S.*

Three months is the time usually allowed to prepare for the L. O. S. After attending twenty confinements, you may present yourself for the society's examination, and, if you pass, gain their certificate. The City of London Lying-in Hospital, City Road, E.C., the British Lying-in Hospital, Endell Street, W.C., and the East End Mothers' Home, 396 Commercial Road, E., all offer rapid and cheap courses of instruction.

*Open-Air Training.*

(7) Would you kindly tell me of any sanitarium in England or Germany where I could be trained as a nurse for the open-air treatment of consumption? I hope ultimately to take up private work in this direction?—*M. V. I.*

Presuming that you are a trained nurse (your letter being dated from a hospital), your best plan would be to advertise. Many medical men are opening private sanatoria in England, and will need nurses.

*America.*

(8) I want to train as a nurse. As, however, I intend living in U.S.A., would you kindly tell me if it (*a*) would be better to train in a London hospital first or (*b*) to enter an American school? 2. Would I obtain any information as to American hospitals in "Burdett's Directory?" 3. Is "How to Become a Nurse," by Miss H. Morten, the most recent publication on the subject?—*M. H.*

1. It would be more advantageous to be trained in the country in which you intend to live. 2. Yes, full information of the largest. 3. No. "The Nursing Profession: How and Where to Train" is the latest.

*Superfluous Hair.*

(9) Can anyone tell me if electrolysis is the only means of removing superfluous hair? If so, where is this treatment performed?—*G.*

Consult a skin specialist, as in the hands of an unskilful operator the cure is worse than the disease, the pits left by the electric needle being more disfiguring than the hair.

*District Work.*

(10) Can you tell me whether, if I went in for maternity training, I could obtain a post as district nurse without certificate for general training?—*Nurse Mildred.*

No. The Queen's nurses are required to have two years' certificate at least from a general hospital, and they then are given an additional six months' course of instruction in district work.

*Epileptic Fits.*

(11) Can you tell me of a home where a boy of nine could be admitted? He takes three or four epileptic fits a day. His father is a working man and has four other children.—*District Nurse.*

The Home for Epileptics, Maghull, near Liverpool (address Hon. Secretary, 2 Exchange Street East, Liverpool), receives third-grade patients at 7s. 6d. a week. The Epileptic Colony, Chalfont St. Peter's (office, 12 Buckingham Street, Strand, W.C.), receives patients at 10s. a week (subject to alteration by committee). There is a ward for children at the National Hospital for the Paralysed and Epileptic, Queen's Square, Bloomsbury, W.C.

*Nursing.*

(12) To which of the London hospitals can a lady go to learn some nursing on the payment of a fee?—*Inquirer.*

The London Hospital, E., is one of several, but the duties there are heavy. See others in Burdett's "Nursing Profession: How and Where to Train."

*Johns Hopkins Hospital.*

(13) Will you tell me if the Johns Hopkins Hospital, Baltimore, U.S.A., employs other than the nurses trained there? What are the terms of training, and what chance would an English trained nurse have of getting an appointment there?—*E. P. B.*

We cannot tell you the rules relating to nursing appointments at the Johns Hopkins Hospital. Apply for training to the Principal of the Training School. After a personal interview, probationers are received for two months' trial and afterwards for three years' training. Premium *nil.* Salaries during training *nil.* Scholarships, however, are open for competition.

*Convalescent Home.*

(14) Can you kindly tell me of a convalescent home where a young girl suffering from nervous exhaustion can be placed?—*Paris.*

Consult Burdett's "Hospitals and Charities," or the "Englishwoman's Year Book."

*Revolving Shelters.*

(15) Since writing about the above shelters, I have found an old *Hospital* with a description of one that suits my patient in every way, made by Messrs. Boulton & Paul, Norwich. They tell me, however, that there is no way of shutting it up at night or in wet weather. Can you tell me if this is usual, or are there other makers to whom I can go?—*R. W.*

It is desirable to expose the places used by consumptives to the air as much as possible. The open side can always be turned away from the wind. If closed it would cease to be "open-air."

*Private Coaching.*

(16) Will you kindly tell me where I could be privately coached for a hospital examination at a moderate fee?—*R. M.*

Miss Lamport, 52 St. John's Wood Road, Regent's Park, N.W., makes a speciality of this work.

*Stewardess.*

(17) I should like to know if the salaries earned by stewardesses on the liners are good, and what are the duties? I have been a nurse for the last three years, but am not certificated.—*Sailor.*

Some months ago a similar query brought replies from a number of correspondents giving anything but a rosy description of the position of a stewardess. The salary is fair, and is largely increased by passengers' "tips." A personal introduction is usually needed to get an appointment, and, of course, the personal comfort of the stewardess depends upon the ship she sails in and the passengers on whom she attends.

*Paralytic Child.*

(18) Can you tell me of a home where a child of four, suffering from paralysis, would be taken for a small payment?—*Nurse D.*

The Hospital and Home for Incurable Children, 2 Maida Vale, W., would suit your patient admirably if there is a vacancy.

*Private Work Abroad.*

(19) 1. I would like to take a post of matron in a cottage hospital. Can you tell me the name of some good book bearing on a matron's duties, such as office work, housekeeping, the ordering of a large staff, &c. 2. I want to go to the Riviera or to Cairo this winter, either in charge of a patient or to take up private work. Will you tell me what chance of success I have?—*Garde Molade.*

The Secretary of the School of Housewifery and Domestic Science, 101 and 105 Stamford Hill, W., would be able to direct your studies in household management, whilst Miss Orme's "The Matron's Course" or Miss Lückes' "Hospital Sisters and their Duties" would help you in training nurses. You should, however, have practical experience gained as assistant matron before undertaking such an onerous post. 2. A nurse's prospect of getting work is good in Cairo during the season. Residential expenses are, however, heavy when without a case. Write to Miss Cutler, Victoria Nursing Home, Cairo. Advertise in medical journals for a travelling patient.

*Hypnotism.*

(20) Has hypnotism been really successful as an anæsthetic or in the cure of disease? Does a person hypnotised remain under the influence of the hypnotiser, or does the control cease after the operation?—*Science.*

Undoubtedly people can be rendered anæsthetic by hypnotism, but as to the cure of disease that is quite another matter. When the hypnotic condition passes off the performance is over. But, and this is a very important point, the patient who has once been hypnotised is comparatively easily hypnotised again, and unfortunately sometimes has a craving for a repetition of the process.

*Nurses' Agency.*

(21) Having had 12 years' experience in organising and managing our district Nurses' Association, and 25 years as secretary of our local dispensary, I should now like to turn this experience to pecuniary advantage. Could you kindly tell me if it be feasible to begin a nurses' "agency," and also the amount of fees usually charged by similar institutions?—*S. A. B.*

Your experience and connection give you many advantages in the kind of work you contemplate. A nurses' agency is, however, a novel departure, and consequently you will have to feel your way before launching out. You must regulate your fees within such limits as will provide a living for yourself and the sum your clients can afford. In domestic and scholastic agencies it is usual to charge a preliminary fee to cover booking and postage expenses, and then from $7\frac{1}{2}$ per cent. upwards from employer or employed, or from both, upon the first year's salary, when the engagement is made. Your advertisement is comprehensive.

### Standard Books of Reference.

"The Nursing Profession: How and Where to Train." 2s. net; post free, 2s. 4d.

"The Nurses' Dictionary of Medical Terms." 2s.

"Burdett's Series of Nursing Text-Books." 1s. each.

"A Handbook for Nurses." (Illustrated.) 5s.

"Nursing: Its Theory and Practice." New Edition. 3s. 6d.

"Helps in Sickness and to Health." Fifteenth Thousand. 5s.

"The Physiological Feeding of Infants." 1s.

"The Physiological Nursery Chart." 1s.; post free, 1s. 3d.

All these are published by THE SCIENTIFIC PRESS, Ltd., and may be obtained through any bookseller or direct from the publishers, 28 and 29 Southampton Street, London, W.C.

